# Measurement of Walking Ground Reactions in Real-Life Environments: A Systematic Review of Techniques and Technologies

**DOI:** 10.3390/s17092085

**Published:** 2017-09-12

**Authors:** Erfan Shahabpoor, Aleksandar Pavic

**Affiliations:** 1 Department of Architecture and Civil Engineering, University of Bath, Claverton Down, Bath BA2 7AY, UK; 2 INSIGNEO Institute for In-Silico Medicine, Department of Civil & Structural Engineering, University of Sheffield, Sir Frederick Mappin Building, Sheffield S1 3JD, UK; 3 College of Engineering, Mathematics and Physical Sciences, University of Exeter, North Park Road, Exeter EX4 4QF, UK; a.pavic@exeter.ac.uk

**Keywords:** walking gait analysis, kinematics, joint kinetics, boundary condition, ground reaction moments, optimization, indeterminate closed-kinematic chain

## Abstract

Monitoring natural human gait in real-life environments is essential in many applications, including quantification of disease progression, monitoring the effects of treatment, and monitoring alteration of performance biomarkers in professional sports. Nevertheless, developing reliable and practical techniques and technologies necessary for continuous real-life monitoring of gait is still an open challenge. A systematic review of English-language articles from scientific databases including Scopus, ScienceDirect, Pubmed, IEEE Xplore, EBSCO and MEDLINE were carried out to analyse the ‘accuracy’ and ‘practicality’ of the current techniques and technologies for quantitative measurement of the tri-axial walking ground reactions outside the laboratory environment, and to highlight their strengths and shortcomings. In total, 679 relevant abstracts were identified, 54 full-text papers were included in the paper and the quantitative results of 17 papers were used for meta-analysis and comparison. Three classes of methods were reviewed: (1) methods based on measured kinematic data; (2) methods based on measured plantar pressure; and (3) methods based on direct measurement of ground reactions. It was found that all three classes of methods have competitive accuracy levels with methods based on direct measurement of the ground reactions showing highest accuracy while being least practical for long-term real-life measurement. On the other hand, methods that estimate ground reactions using measured body kinematics show highest practicality of the three classes of methods reviewed. Among the most prominent technical and technological challenges are: (1) reducing the size and price of tri-axial load-cells; (2) improving the accuracy of orientation measurement using IMUs; (3) minimizing the number and optimizing the location of required IMUs for kinematic measurement; (4) increasing the durability of pressure insole sensors, and (5) enhancing the robustness and versatility of the ground reactions estimation methods to include pathological gaits and natural variability of gait in real-life physical environment.

## 1. Introduction

Human gait analysis encompasses the quantification and interpretation of measurable walking or running gait parameters, and is a crucial tool in many applications including medical diagnosis and treatment, professional sports and wearable robotics [1]. Gait analysis conventionally involves direct measurement of body motion (kinematics) and boundary conditions (ground reaction forces GRF(t) and moments GRM(t) or alternatively GRF(t) and the plantar centre of plantar pressure CoP(t) under each foot). The joint forces and torques (kinetics) and muscle forces can subsequently be estimated from these measured data. The spatiotemporal gait metrics, such as stride length, joint angles, peak GRF(t) and inter-segmental forces provide important measures of the health and performance of the gait [2]. Therefore, effective techniques and technologies are required to measure the gait parameters accurately and realistically for different applications, tasks and population cohorts.

Currently, a combination of an optical motion capture system and a pair of floor mounted forceplates or an instrumented treadmill are being used as ‘gold-standard’ setup to measure body kinematics and GRF(t), GRM(t) and CoP(t) signals, respectively, in laboratory setting [3,4,5]. However, measuring gait using these equipment in the laboratory environment entails several limitations and drawbacks:

- Quantification of the spatiotemporal gait fluctuations over time or due to environmental, behavioural or contextual factors are essential in many applications such as understanding the motor control of gait, quantifying pathologic and age-related alterations in the locomotor control system, and augmenting objective measurement of mobility and functional status [6]. However, It is shown that measuring a limited number of strides in the gait laboratory may not represent natural cycle-by-cycle gait variations [7].
- Recent studies showed that subjects may modify their gait inside laboratory environment and may mask or exaggerate their problem during the test [8].
- The standard two-forceplates setup used in biomechanics laboratories makes it possible to measure ground reactions for only one step and enforces a limited area for foot placement, which can alter the natural gait [9,10,11,12,13,14,15]. The instrumented treadmills, on the other hand, can record continuous walking/running of a test subject [10,12]. However, they can only record the ground reactions while subject is moving in a straight line with a constant speed [16].
- The standard gait laboratory equipment (optical motion capture, force plates and instrumented treadmills) are very expensive and cumbersome and require expertise to operate [16]. These factors restrict their availability to a limited number of well-equipped gait laboratories.
- Long-term measurement of gait in real-life environment is essential in many applications including quantification of disease progression [17], monitoring the effects of treatment [18], and monitoring alteration of performance biomarkers in professional sports [19,20]. Realistic monitoring of the dynamic gait variations can signify disease severity and medication utility, and can be used to document quantitatively improvements in response to therapeutic interventions, significantly more effective than what can be learned based on the average, typical stride measured in a laboratory [6].

To overcome the abovementioned limitations of gait measurement in the laboratory environment, and to realise the ample potentials of gait monitoring in real-life environment, several techniques and technologies have been developed in the past three decades to measure or estimate tri-axial GRF(t) and CoP(t) signals using wearable sensors. This paper systematically reviews such techniques and technologies in a chronological order, analyses their strengths and shortcomings and suggests promising avenues for their future development. The reviewed techniques are categorized in three categories based on the type of measured parameters:

- Methods based on measured kinematic data use a human body dynamic model to estimate GRF(t), GRM(t) and/or CoP(t) signals from acceleration of different body segments. This category of techniques can potentially use the inexpensive and durable wearable Inertial Measurement Units (IMUs) for measuring body kinematics and therefore are potentially practical. Although these methods are prone to IMU errors in orientation measurement and are sensitive to the characteristics of the body dynamic model, they have shown competitive accuracy of 54 N, 33 N and 10 N root-mean-square-error (RMSE) (assuming an average subject weight of 750 N) for estimating $GRF_{v}\left(t\right),\;GRF_{ap}\left(t\right)$ and $GRF_{ml}\left(t\right)$, respectively.
- Methods based on measured plantar pressure use a matrix of insole pressure sensors to measure plantar pressure of each foot perpendicular to the contact surface. A computational method is usually used to estimate tri-axial GRF(t) and plantar CoP(t) signals. Although current pressure insole sensors show limited durability and high sensitivity to their boundary condition in the shoe, this category of method have shown to achieve competitive average accuracy of 61 N, 25 N and 12 N RMSE for estimating $GRF_{v}\left(t\right),\;GRF_{ap}\left(t\right)$ and $GRF_{ml}\left(t\right)$, respectively.
- Methods based on force measurement directly measure tri-axial GRF(t) and CoP(t) signals under each foot using a pair of shoes instrumented with tri-axial force sensors and IMUs. Although the cumbersome electromechanical form factor of these systems can affect the natural gait and reduce its practicality, this class of techniques is shown to achieve the highest average accuracy of 13 N, 13 N and 10 N RMSE for estimating $GRF_{v}\left(t\right),\;GRF_{ap}\left(t\right)$ and $GRF_{ml}\left(t\right)$, respectively.

The study selection and data analysis protocol used in this review are discussed in detail in Section 2. Section 3 of this paper reviews the methods that use measured kinematic data to estimate tri-axial GRF(t), GRM(t) and/or CoP(t) signals. The techniques that estimate GRF(t), GRM(t) and/or CoP(t) using measured plantar pressure are discussed in Section 4. The methods that directly measure GRF(t), GRM(t) and/or CoP(t) using wearable forceplates are reviewed in Section 5. The accuracy and practicality of the reviewed techniques and technologies are compared in Section 6. Finally, conclusions are highlighted in Section 7 and a few directions for future research in this area are outlined.

## 2. Data Analysis

**Figure 1:**
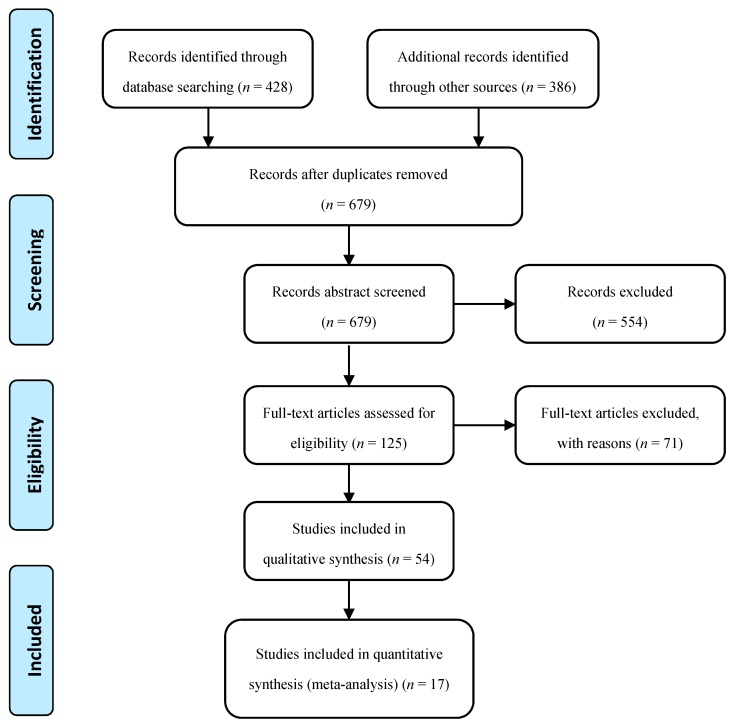
Study selection through the different phases using PRISMA framework [21].

A systematic literature review was carried out based on the PRISMA scheme [21], with a pre-planned meta-data analysis protocol, inclusion and exclusion criteria, and data reporting framework (Figure 1). Only English-language peer-reviewed papers proposing both the technique and technology for estimation or measurement of more than one component of tri-axial walking ground reaction forces GRF(t), tri-axial walking ground reaction moments GRM(t) and plantar centre of pressure CoP(t), were considered for this review. Furthermore, only the studies with quantitative experimental validation of the proposed method were selected for meta-analysis. Studies with measurement technologies that potentially could not be used outside the laboratory environment, such as motion capture systems, forceplates and treadmills were not included in this review.

In total, 679 relevant abstracts were identified from scientific databases including Scopus, ScienceDirect, Pubmed, IEEE Xplore, EBSCO and MEDLINE (Figure 1). The search terms used included ground reaction force, walking force, centre of pressure, plantar pressure, wearable sensors, inertial measurement unit, IMU, force sensor, outdoor measurement and walking gait. Last search date was 6 November 2016.

A set of 125 full-text articles were assessed for eligibility and 54 full-text papers were deemed to meet the criteria to be included in the review. Information from each article were summarized in a pre-designed table, containing information (where available) on: study design, instrument/sensor type and specifications, sensor placement on body, measurement protocol, number of test subjects, subjects’ anthropometric data, details of the technique used to measure or estimate GRF(t), GRM(t) and CoP(t) signals, validation protocol, and corresponding errors in the methods results. Root-mean-square-error (RMSE) was found the most common reported parameter to compare the accuracy of the results of the reviewed techniques. Only 17 papers were found to have RMSE data reported for their proposed methods and, therefore, these papers were used for meta-analysis and comparison.

The reviewed techniques are classified into three groups for analysis based on their measured sensory inputs: (1) methods based on measured kinematic data (Section 3); (2) methods based on measured plantar pressure (Section 4); and (3) methods based on directly measured ground reactions (Section 5). The *accuracy* and *practicality* of each proposed technique and technology for real-life continues measurement were analysed separately for each class and then cross-compared in Section 6.

## 3. Methods Based on Measured Kinematic Data

This class of methods uses measured body kinematic data (i.e., three-dimensional orientation, tri-axial linear and rotational displacement, velocity and acceleration of each body segment) combined with a dynamic human body model to estimate GRF(t) and CoP(t). The joint torques and forces and the ground reactions are estimated using Inverse dynamics (ID) method [22]. As this class of methods only relies on kinematic measurement and anthropometric data, it eliminates the need for a forceplate/treadmill and therefore, makes it possible to collect the required kinematic data outside the laboratory environment using, for example, wearable IMUs [23]. However, in contrast with other classes of methods, the inherent dependency of this class of methods on a body dynamic model can introduce uncertainty in estimated results.

### 3.1. Double-Support Indeterminacy

In the context of biomechanics, the ID method is usually used to estimate the joint forces and torques responsible for an observed motion (kinematics) following a set of assumptions [24,25]:

- The joints are frictionless pin-joints;
- The body segments are assumed to be rigid, with their mass concentrated at their centres of mass;
- The co-contraction of agonist and antagonist muscles are neglected;
- The air friction is assumed to be negligible.

A link-segment model of the human body is typically used to model the interconnected body limbs and joints. In the ID method, the human body’s measured kinematic data, the inertial properties of body segments (mass and moment of inertia) and boundary conditions such as GRF(t) and GRM(t) signals are used as input. ID analysis conventionally starts with the measured ground reactions and, beginning with those limbs in contact with the ground, calculates joint forces and torques successively from distal to proximal limbs [22]. However, to estimate the tri-axial GRF(t) and GRM(t) signals from kinematic data, ID simulation needs to be carried out in reverse order from proximal to distal limbs.

During the single-support phase (SSP) of the gait, tri-axial GRF(t) and GRM(t) of the foot in contact with the ground (stance foot) can easily be calculated using Newton-Euler equations and ID simulation [22]. For each body segment ‘*i*’ with mass $m_{i}$, the second moment of inertia $I_{i}$ and linear $a_{i}$ and rotational $\alpha_{i}$ accelerations with respect to its centre of mass, the total GRF(t) and GRM(t) signals in the x, y and z axes can be calculated using Equations (1)–(6) [25]:

(1) $$\sum^{\text{​}}GRF_{x}\left(t\right)=\sum_{i}(m_{i}\times a_{i,x}\left(t\right)),$$

(2) $$\sum^{\text{​}}GRF_{y}\left(t\right)=\sum_{i}(m_{i}\times a_{i,y}\left(t\right)),$$

(3) $$\sum^{\text{​}}GRF_{z}\left(t\right)=\sum_{i}(m_{i}\times a_{i,z}\left(t\right)),$$

(4) $$\sum^{\text{​}}GRM_{x}\left(t\right)=\sum_{i}(F_{i}\left(t\right)\times r_{i,x}\left(t\right)+I_{i,xx}\times \alpha_{i,x}\left(t\right)),$$

(5) $$\sum^{\text{​}}GRM_{y}\left(t\right)=\sum_{i}(F_{i}\left(t\right)\times r_{i,y}\left(t\right)+I_{i,yy}\times \alpha_{i,y}\left(t\right)),$$

(6) $$\sum^{\text{​}}GRM_{z}\left(t\right)=\sum_{i}(F_{i}\left(t\right)\times r_{i,z}\left(t\right)+I_{i,zz}\times \alpha_{i,z}\left(t\right)),$$

where $r_{i}$ is the perpendicular distance between the centre of mass of segment ‘*i*’ and the point of reference for the calculation of GRM(t). In Equations (1)–(6), the coordinate axes of the body-attached reference frame are chosen to be the principal axis of inertia, and gyroscopic effects are assumed to be negligible.

The main challenge of using the ID method to estimate the GRF(t) and GRM(t) signals, however, occurs in the double-support phase (DSP), where both legs are in contact with the ground and, therefore, the body link-segment system forms an indeterminate closed kinematic chain with more unknowns than equations of motion. The methods presented in Section 3.2 deal mainly with this challenge and propose methods to estimate tri-axial GRF(t) and GRM(t) or CoP(t) signals during DSP.

To provide a comparative measure of the level of accuracy achievable by this class of methods when estimating tri-axial GRF(t) during the SSP and DSP, Oh, et al. [26] reported the RMSE of 0.352 N/kg, 0.071 N/kg and 0.051 N/kg for estimating $GRF_{v}\left(t\right),\;GRF_{ap}\left(t\right)$ and $GRF_{ml}\left(t\right)$, respectively, during SSP and 0.787 N/kg, 0.437 N/kg and 0.168 N/kg for estimating $GRF_{v}\left(t\right),\;GRF_{ap}\left(t\right)$ and $GRF_{ml}\left(t\right)$, respectively, during the DSP. This shows a considerably higher accuracy of the estimated ground reactions during SSP compared with DSP.

### 3.2. Methods

To solve the indeterminacy problem of DSP, Quanbury and Winter [27] and Robertson and Winter [28] suggest extrapolating the GRF(t) signals during DSP by fitting cubic polynomials to the known force values at the beginning and end of the SSP for each foot. A cubic spline was fitted to a set of points derived from heel, ankle and toe kinematics to estimate CoP(t). They reported a mean error of 18% and 14% for the vertical ground reaction force $GRF_{v}\left(t\right)$ and CoP(t). However, the error value for the anterior-posterior ground reaction force $GRF_{ap}\left(t\right)$, 38%, was less promising.

McGhee, et al. [29,30], McGhee [31], Hardt and Mann [32] and Morecki, et al. [33] suggested a linear function of DSP time to approximate the transfer of GRF(t) and GRM(t) between the trailing and leading foot. Hardt and Mann [32] used a five segment human body model (shanks, thighs and torso) and estimated the total GRF(t) and GRM(t) signals for three subjects, using measured kinematic data (Equations (1)–(6)). They suggested the dependence of GRF(t) and GRM(t) in the right and left foot as:

(7) $$\vec{GRF_{total}}\left(t\right)=\vec{GRF_{left}}\left(t\right)+\vec{GRF_{right}}\left(t\right),$$

(8) $$\vec{GRM_{total}}\left(t\right)=\vec{GRM_{left}}\left(t\right)+\vec{GRM_{right}}\left(t\right),$$

(9) $$\vec{GRF_{left}}\left(t\right)=\frac{t-t_{1}}{t_{2}-t_{1}}\vec{GRF_{total}}\left(t\right),$$

(10) $$\vec{GRM_{left}}\left(t\right)=\frac{t_{2}-t}{t_{2}-t_{1}}\vec{GRM_{total}}\left(t\right),$$

where, $t_{1}$ and $t_{2}$ are the beginning and end time of DSP, respectively, measured using footswitches on the heel and toe of the subjects’ shoe. They reported maximum error values of 8% and 15% for the estimated $GRF_{v}\left(t\right)$ and CoP(t), respectively, but concluded that the linear hypothesis (Equation (9)) was inappropriate for $GRF_{ap}\left(t\right)$ with a maximum error value of 49% [34]. Their model only considered the lower limbs and was only applied to three healthy subjects, with no experimental validation with force plate data.

Vaughan, et al. [34] suggested a more generic approach to solve different types of closed-loop indeterminacy problems. They used a 14 rigid segments model of the human body (head, upper arms, forearms, hands, thighs, shanks, feet and trunk) to formulate the Newton-Euler equations of motion of the body, where GRF(t) and GRM(t) signals corresponding to each foot were included as boundary conditions. Only motion in the sagittal plane was considered in their study. A cost function, defined as the weighted sum of joint forces and torques, was minimised in the optimisation process to find the unknown joint forces and torques and GRF(t) and GRM(t) signals.

The method was applied to three closed-loop problems: walking up the stairs, vertical jumping and cartwheeling for a healthy test subject (height: 1.75 m, mass: 70.99 kg). Kinematic data were measured using a Locam motion capture system and GRF(t) signals were measured using a Kistler tri-axial force plate (Kistler Instrumente AG, Winterthur, Switzerland). Vaughan, et al. [34] reported the mean peak-to-peak error of 11%, 11% and 38% in the estimated $GRF_{v}\left(t\right)$, $GRF_{ap}\left(t\right)$ and $CoP_{ap}\left(t\right)$, respectively, for walking up the stairs (closest activity to ambulation). Their model did not need identification of DSP start and end points and is applicable to different closed-loop problems. However, a very limited experimental validation was provided and only 2D movement was considered.

**Figure 2:**
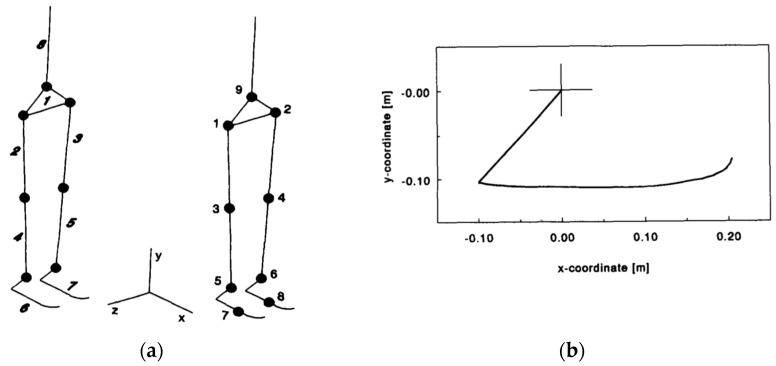
The eight segments human body model (**a**) with experimentally measured foot shape; (**b**) used by Koopman et al. [35].

Koopman, et al. [35] used an eight segment (feet, shanks, thighs, pelvis and torso) human body model for ID simulation, featuring an experimentally measured foot shape (Figure 2) to estimate the total GRF(t) and GRM(t). CoP(t) was defined as the point of contact of the adducting foot model with ground. Koopman, et al., proposed a ‘shift’ function to define the transfer of body weight from the trailing foot to the leading foot during DSP, but no explanation about this function was provided. An optimisation process was used to estimate GRF(t) and GRM(t) signals corresponding to each foot by minimising the joint torques.

In the experiment above, a set of measurement was carried out on a healthy male subject (age: 24 years; weight: 83 kg; height: 1.83 m) where the subject walked for 25 m in a straight line at a comfortable speed. Six goniometers were used to measure the hip, knee and ankle flexion of both legs, and two footswitches were used under each foot to determine heel-contact and toe-off time. No GRF(t) and GRM(t) measurements were carried out and, therefore, no numerical validation was provided. Koopman, et al., reported that results were very sensitive to the realistic shape of the foot model, accurate measurement of joint angles and the level of noise.

Audu, et al. [36] used a full-body 10 segment, 17 degrees of freedom (DOFs) three-dimensional biomechanical model of a human to formulate the ID equations of motion and estimate GRF(t) and CoP(t) during standing. Indeterminacy of the closed-chain model was resolved using a static optimisation method, by minimising the sum of the joints torques for any given posture. This model was later validated [37] against a set of experimental data collected from four healthy male subjects (age: 23–26 years, height: 1.61–1.78 m; weight: 58.5–86.6 kg) standing still on two force platforms with different postures. Kinematic data were collected using an optical motion capture system. Results of the model showed mean peak-to-peak errors of 15.6%, 13.6–24.6%, 7.4% for $GRF_{v}\left(t\right)$, $GRF_{ap}\left(t\right)$ and $GRF_{ml}\left(t\right)$, respectively, and 23.2% for $CoP_{ap}\left(t\right)$. However, no evidence was provided on the performance of the model in a dynamic case such as walking.

**Figure 3:**
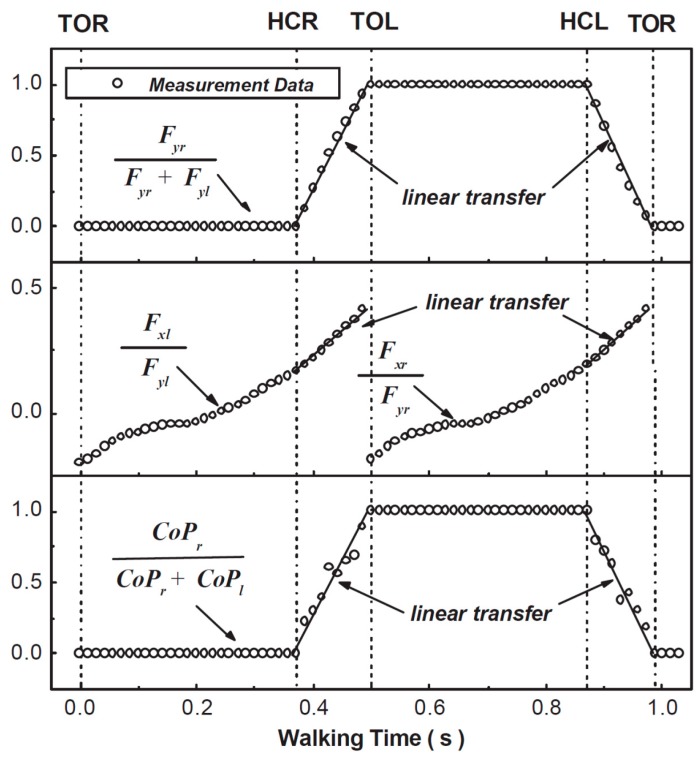
Measured and modelled (linear) transfer ratios of vertical (*y*) and anterior-posterior (*x*) ground reaction forces (*F*) and *CoP* between left (*l*) and right (*r*) foot suggested by Ren, et al. [38].

Ren, et al. [38,39] used a 3D full-body model comprising 13 rigid segments (head, torso, pelvis, upper arms, forearms, thighs, shanks and feet) for ID analysis over the complete gait cycle. The anatomical coordinate systems of body segments were mainly adapted from Cappozzo, et al. [40] and van der Helm and Pronk [41]. The tri-axial GRF(t) and GRM(t) signals during SSP were directly calculated using Newton-Euler equations (Equations (1)–(6)). To solve the indeterminacy problem of the closed-chain model, Ren, et al. [38] proposed a linear transfer relationship in the sagittal plane, assuming that (Figure 3):

- The ratio of the $GRF_{v}\left(t\right)$ on the heel-strike foot to the total $GRF_{v}\left(t\right)$ varies linearly during the DSP (Figure 3, top).
- The ratio of the $GRF_{ap}\left(t\right)$ to the $GRF_{v}\left(t\right)$ on the toe-off foot varies linearly during the DSP (Figure 3, middle).
- The ratio of CoP(t) for the heel-strike foot to the sum of the CoP(t) for both feet varies linearly during the DSP (Figure 3, bottom).

A set of full-body kinematic measurements were carried out using an optical motion capture system on two healthy male subjects while they were walking in the laboratory at a comfortable speed. However, no force measurement was carried out and, therefore, no validation was presented.

Later in 2008, Ren et al. [39] improved their model and proposed the ‘smooth transition assumption’ (STA), based on the trend of change of measured force plate data for each foot:

- GRF(t) and GRM(t) signals of the trailing foot reduce smoothly to zero during the DSP.
- The ratios of GRF(t) signals to their values at contralateral heel strike (i.e., the non-dimensional ground reactions) can be expressed as functions of DSP duration (termed transition functions).

The following semi-empirical transition functions were then proposed to estimate GRF(t) and GRM(t) signals:

(11) $$\{\begin{array}{c} \frac{GRF\left(t\right)}{GRF_{0}}=e^{-{\left(t/T_{ds}\right)}^{3}}\\ \frac{GRM\left(t\right)}{GRM_{0}}=e^{-{\left(t/T_{ds}\right)}^{3}} \end{array}$$

(12) $$\frac{GRF_{ap}\left(t\right)}{GRF_{ap0}}=\left(k_{1}e^{-{\left[\left(t-t_{p}\right)/T_{ds}\right]}^{2}}-k_{2}\frac{t}{T_{ds}}\right)$$

where, $GRF_{0}$ is the corresponding GRF(t) magnitude at the contralateral heel strike, $T_{ds}$ is half of the DSP duration, *t_p_* = 2$T_{ds}$/3 is the peak force time and $k_{1}$ and $k_{2}$ are empirical constants. In each gait cycle, GRF(t) of the trailing foot was calculated using the transition functions and then Newton-Euler equations were used to calculate the force on the leading foot. Subsequently, the same process is repeated to calculate GRM(t) signals.

**Figure 4:**
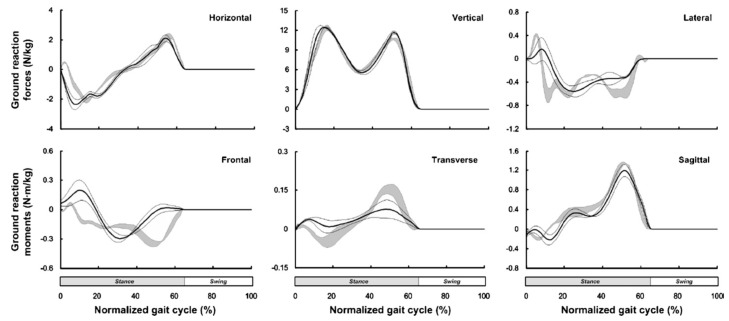
Comparison of the calculated (solid lines) and measured (shaded area) GRF(t) and GRM(t) signals normalized by the body weight for a typical test subject walking at normal speed (1.5 ± 0.28 m/s). Results are in the form of mean ± one standard deviation. (After Ren, et al. [39]).

The proposed model was then verified against a set of gait measurements with three healthy male subjects (age: 31 ± 3.6 years, weight: 76.3 ± 7.5 kg, height: 1.79 ± 0.09 m). An optical motion capture system and two forceplates were used to measure reference motion, GRF(t) and GRM(t) data, respectively. The model proposed by Ren, et al. [39] showed good results in the sagittal plane, with normalised root-mean-square-error (NRMSE) of 6% for $GRF_{v}\left(t\right)$, 10% for $GRF_{ap}\left(t\right)$ and 13% for the GRM(t). The results, however, were less promising in the frontal and transverse planes (Figure 4). The main sources of error were identified as skin movement artefacts and errors in the anthropometric parameters.

The method proposed by Ren, et al. [39], however, was based on an empirical set of transition functions derived from a limited set of experimental data and, therefore, might not be applicable to pathological cases. Moreover, the method was based on the assumption of symmetrical motion of the right and left foot that might not hold true in some cases.

Winiarski and Rutkowska-kucharska [42] carried out a set of gait experiments involving fifty three male (age: 31.5 ± 9.7 years) and thirty three female (age: 33.9 ± 10.9 years) subjects while walking at their preferred speed. In each test, kinematic data were measured using 18 passive markers and a video based motion capture system, and tri-axial GRF(t) and GRM(t) signals were measured using a force plate. The Clauser, et al. [43] model was used to estimate the trajectory of the body centre of mass (CoM) from kinematic data using regression equations [44,45]. The CoM trajectory was then double-integrated and multiplied by the body mass to estimate the GRF(t) signals. They suggested that the proposed methodology is sensitive to errors in anthropometry and marker placement, and requires a complex 3D motion analysis system.

Lugris, et al. [46] and Cuadrado, et al. [47] proposed a foot-ground contact model (FCM) to estimate each foot GRF(t) and GRM(t) signals during DSP. A set of 3D kinetic and kinematic data were collected from a healthy male subject (age: 37, mass: 74 kg and height: 180 cm) using two force plates and an optical motion capture system. A 3D 18 segment model of a human body with 57 degrees of freedom was used for ID simulation.

Kinematic data were initially used in Equations (1)–(6) to calculate the total GRF(t) and GRM(t) signals. The parameters of the proposed FCM for both feet were considered as the design parameters of an optimisation process. The difference between the total GRF(t) obtained using kinematics data and FCM was defined as the cost function and was minimised to find each foot GRF(t) and GRM(t) signal.

**Figure 5:**
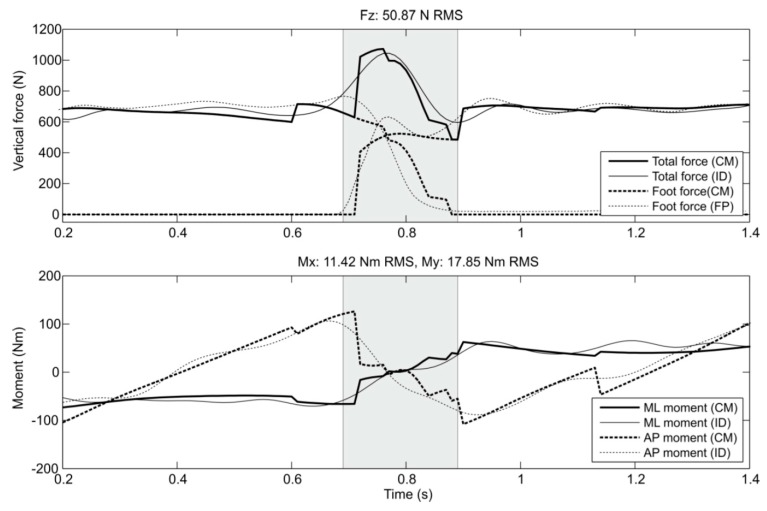
Comparison of the vertical force and the medial-lateral (ML) and anterior-posterior (SP) moments obtained from FCM (CM) and ID with forceplate (FP) measurements (after [46]).

Results of the proposed method showed a reasonable agreement with the force plate measurements (Figure 5). However, very limited experimental data (from only one person) were used in the study and no quantitative validations were presented. The analysis was further limited to the vertical component of GRF(t). The implementation of FCM was shown to be computationally demanding and, therefore, not suitable for real-time/wearable applications.

Choi, et al. [48] suggested an Artificial Neural Network (ANN) model to solve the indeterminacy problem during DSP. ANN is a very flexible and strong tool for non-linear modelling and, thus, can be particularly useful in model-based gait analysis [49]. A set of posture, gait and asymmetrical movement experiments was carried out on 13 healthy adults. The VICON motion capture system [50] and two AMTI force platforms [51] were used to measure the kinematic data and ground reactions, respectively. Newton-Euler equations (Equations (1)–(6)) were used to calculate GRF(t) during SSP.

**Figure 6:**
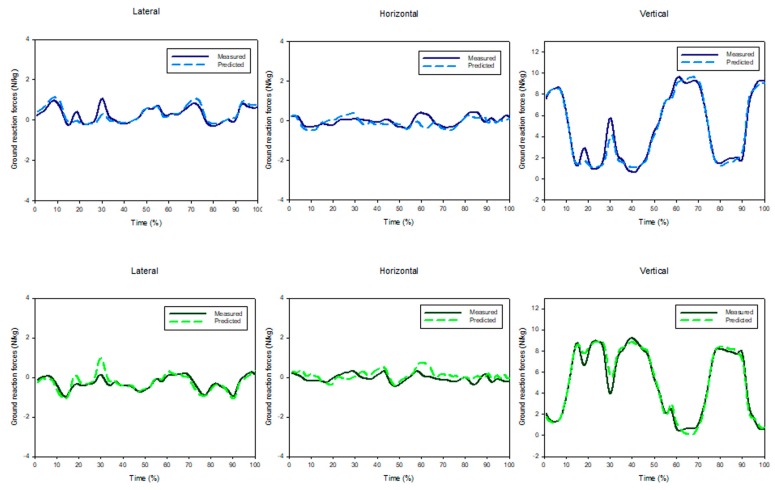
The measured and estimated GRF(t)s of a typical subject normalized by body mass (after [48]).

A three-layer feed-forward ANN model (an input layer, a hidden layer and an output layer) was considered to predict three components of GRF(t) pertinent to each foot. Thirteen input parameters were selected for ANN out of the initial set of 1098 candidates, using prior knowledge and application of a self-organising map and genetic algorithm general regression neural network [52]. Twelve data sets were used to train the ANN and the final data set was used to validate the model. Results of the study of Choi, et al., showed an acceptable performance of the ANN model with the correlation coefficients of 0.85, 0.88 and 0.97 between the measured and estimated $GRF_{ml}\left(t\right)$, $GRF_{ap}\left(t\right)$ and $GRF_{v}\left(t\right)$ of each foot, respectively (Figure 6).

In a very similar study, Oh, et al. [26] suggested a method based on ANN to solve the indeterminacy problem during DSP. Some 48 healthy test subjects (28 males and 20 females, age: 25.4 ± 3.1 years; height: 1.72 ± 0.07 m; weight: 66.2 ± 7.5 kg) participated in a series of bare-foot walking tests with their own preferred walking speed. VICON 460 motion capture system [50] and two AMTI forceplates [51] were used to record kinematic data and ground reactions, respectively. A 15 rigid segments (head, thorax, humerus, radius, hands, pelvis, femora, tibiae, and feet) body model and ID (Equations (1)–(6)) were used to find GRF(t) and GRM(t) signals during SSP.

A feed-forward ANN with one input layer, one hidden layer and one output layer was used to find tri-axial GRF(t) and GRM(t) signals during DSP. Data from 43 randomly selected subjects were used to train the ANN, and the remaining five were used for validation of the model. Out of 825 initial candidates, 14 input variables were selected for ANN using prior knowledge, the self-organising map technique and the genetic algorithm general regression neural network process. Cubic spline interpolation was used later to smoothly connect the results of SSP and DSP together.

**Figure 7:**
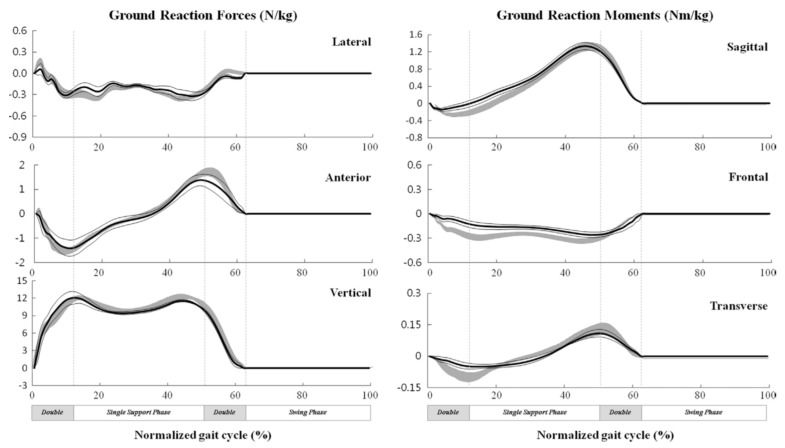
Estimated GRF&Ms normalized by body mass (mean (thick line) ± 1 SD (thin lines), compared with forceplate data (mean ± 1 SD (shaded area)) (after [26]).

The GRF(t) signals pertinent to each foot, predicted by their ANN model, showed a correlation coefficient and peak-to-peak normalised RMSE of 0.918% and 10.9% for $GRF_{ml}\left(t\right)$, 0.985% and 7.3% for $GRF_{ap}\left(t\right)$ and 0.991% and 5.8% for $GRF_{v}\left(t\right)$, respectively (Figure 7). In addition, the correlation coefficients of 0.987 in the sagittal plane, 0.841 in the frontal plane, and 0.868 in the transverse plane were found for GRM(t) signals. Oh, et al., then compared the results of their ANN model with the results of the smooth transition assumption [39] and the foot-ground contact model [46], applied to the same set of experimental data, and found that the ANN results were more accurate. However, Oh, et al. [26] and Choi, et al. [48] ANN models require training data, which might not always be available. These were applied only to healthy subjects and their performance with pathological cases with asymmetric motion is unknown. Finally, ANN is a black-box approach and neither provides any insight about its internal estimation methodology nor gives any link with physical parameters such as kinematic, kinetic and anthropometric parameters and biomechanical concepts.

Robert, et al. [53] suggested more generic methodology to estimate external contact loads on the body using ID simulation and a quadratic optimisation approach [54]. They applied the model to predict the external contact loads during sit-to-stand movements. A whole-body human model made of n_s_ rigid segments was used in the simulation. The model resulted in 6n_s_ linear equations (Newton-Euler) where external contact loads and net joint loads were unknown. Three different cost functions of (1) the external contact forces; (2) the net joint torques and (3) the motor torques normalised by their maximum allowable values were minimised to find unknowns. Although the Robert, et al. [53] method did not require empirical or training data, the contact configurations were simplistic and the method was validated only for sit-to-stand motion.

Fluit, et al. [55] proposed a method to solve the DSP indeterminacy that did not use empirical or training data and is applicable to a variety of activities of daily living (ADLs). Nine healthy subjects (four males and five females; age: 41.6 ± 15.9 years; height: 1.74 ± 0.12 m; weight: 73.0 ± 11.1 kg, body mass index (BMI): 23.9 ± 2.0 kg/m^2^) with no history of musculoskeletal disorders, participated in the study. An optical motion capture system [50] and two custom-built forceplates were used to capture motion data and ground reactions, respectively. Several ADLs were tested, including level walking at comfortable, slow (−30%) and fast (+30%) walking speed, walking over 10 cm, 20 cm and 30 cm obstacles, and stair ascent and descent.

The ID analysis was performed using a 28 DOF full body model, as available in AnyBody Modelling System (version 5.3.1, AnyBody Technology A/S, Aalborg, Denmark) [56]. The mass of body segments was linearly scaled using data from Winter [57]. To solve the DSP indeterminacy problem, Fluit, et al., introduced five artificial muscle-like actuators (one in the vertical direction and two pairs aligned with the medial-lateral and anterior-posterior directions) in their musculoskeletal model, at 12 contact points under each foot. The applied force of each artificial muscle-like actuators and the tri-axial GRF(t) and GRM(t) signals pertinent to each foot were found to be part of the muscle recruitment problem using static optimisation [57].

**Figure 8:**
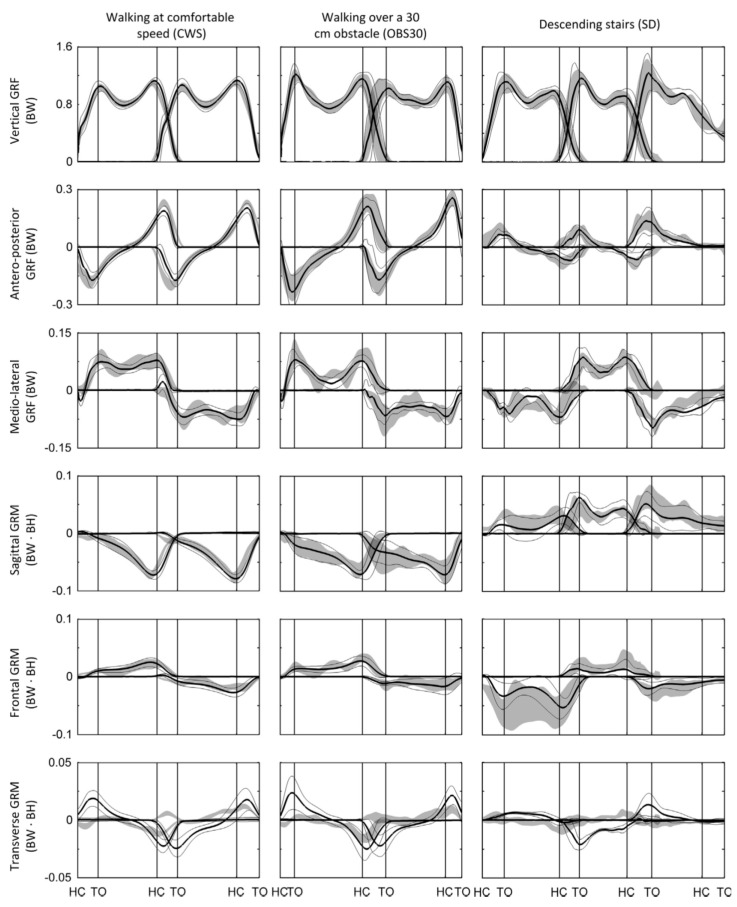
The calculated GRF&Ms normalized to body weight (BW) and height (BH) ±1 standard deviation (SD) around mean (shaded area) compared with the forceplate data (mean (thick line) ± 1 SD (thin lines)). HC: heel contact; TO: toe-off (after [55]).

Results of the simulations of Fluit, et al., showed good prediction of $GRF_{v}\left(t\right)$ (Pearson correlation coefficient *ρ*: 0.621–0.980, median 0.957) and $GRF_{ap}\left(t\right)$ (*ρ*: 0.202–0.969, median 0.957) for most of the activities (Figure 8). The magnitude of $GRF_{v}\left(t\right)$ was slightly but consistently underestimated, whereas the magnitude of $GRF_{ap}\left(t\right)$ was consistently overestimated. The model also showed strong correlations for the sagittal GRM(t) (*ρ*: 0.506–0.922, median 0.789) and frontal GRM(t) (*ρ*: 0.199–0.801, median 0.668). However, weak or sometimes negative correlations were found for the transverse GRM(t) for multiple activities. Fluit, et al., suggested the hinged knee model as the probable source of error in the results.

The Fluit, et al. [55] model, however, is dependent on detailed full-body kinematics and a scaled musculoskeletal model, which must be tailored to each individual for accurate results. Errors due to soft tissue artefacts and muscle activation dynamics were not considered in their model. More importantly, their method demands a very detailed and accurate monitoring of the foot motion, which might not be possible to achieve in an outdoor environment using wearable sensors such as IMUs.

Yang and Mao [58] proposed a seven segment lower-limb model with symmetrical legs to calculate the joint forces and GRF(t) signals in the sagittal plane. Motion of the lower limbs was measured using six two-axis gyroscopes attached to the thighs, shanks and feet and two three-axis accelerometers attached to the feet. Benchmark GRF(t) signals were measured using two force sensors under each foot.

**Figure 9:**
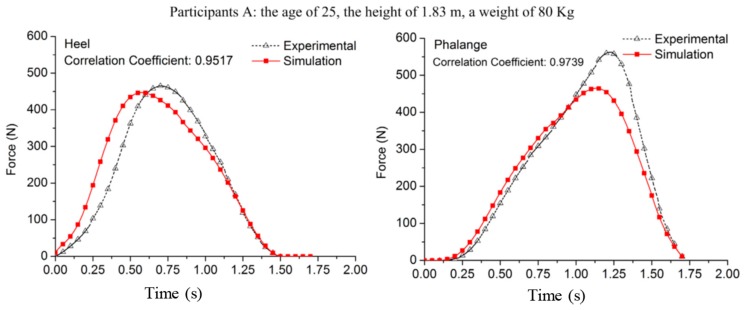
Comparison of the estimated and measured GRFs at heel and phalange locations for a typical test subject (after [58]).

The joint forces were calculated using a forward Newton-Euler formulation. A weighting function was used to estimate the GRF(t) signals at the heel and phalange locations based on a theoretical trajectory of the CoP(t) [59] for each foot. A series of walking tests were carried out on three healthy male subjects (age: 24.5 ± 0.5 years; height: 1.74 ± 0.09 m; weight: 78.3 ± 4.7 kg). The correlation coefficients of 0.9517–0.9958 and 0.9739–0.9958 were reported between the measured and estimated $GRF_{v}\left(t\right)$ signals at the phalange and heel points, respectively (Figure 9). Yang and Mao’s model, however, was developed using a limited experimental data, utilised several simplifying assumptions and was only applied to the sagittal plane.

Later in 2015, Yang and Mao [60] updated their proposed 2D body model to 3D and included the effects of trunk posture variation measured using an IMU in estimating tri-axial GRF(t) and GRM(t) signals. Seven IMUs attached to feet, shanks, thighs and torso were used to measure body kinematics. A set of walking tests was carried out involving two male subjects (age: 24.5 ± 0.5 years; height: 1.67 ± 0.025 m and weight: 68.5 ± 1.5 kg). The reference 3D body motion and the ground reactions were measured using a motion capture system and a bespoke shoe system instrumented with two load cells (LTH400, Futek Advance Sensor Technology, Inc., Irvine, CA, USA) at the phalange and heel of each foot sole, respectively.

3D ID analysis was used to estimate the tri-axial GRF(t) and GRM(t) signals. The timings of the heel-strikes and toe-offs were determined directly from IMU measurements. During DSP, GRF(t) was transferred from the trailing foot to the leading foot using the exponential transfer function suggested by Ren et al. [39] (Equations (11) and (12)). Yang and Mao [60] reported a correlation coefficient of 0.95–0.98 between the measured and estimated $GRF_{v}\left(t\right)$.

### 3.3. Comparison of the Methods

Table 1 compares the absolute and (body) mass normalised RMSE (NRMSE) of the estimated tri-axial GRF(t) and GRM(t) signals for the models suggested by Ren, et al. [39], Lugris, et al. [46], Choi, et al. [48], Oh, et al. [26] and Fluit, et al. [55]. In general, the method suggested by Oh, et al. [26] has the most accurate results, but their model needs subject-specific training. The Fluit, et al. [55] model, on the other hand, has reasonably good accuracy and is not dependent on the training data.

## 4. Methods Based on Measured Plantar Pressure

This class of methods is based on measuring the feet plantar pressure using insole pressure sensors. This pressure is measured only in the axis perpendicular to the plane of the sensor. As a result, in contrast to the force plate measurement, accurate measurement of foot/sensors orientation is also required to transform the pressure data to the global reference coordinate system [61]. In theory, having plantar pressure and sensor orientation data, the vertical $GRF_{v}\left(t\right)$ signals and CoP(t) trajectory can be calculated. However, a mathematical method is required to estimate the anterior-posterior and medial-lateral components of GRF(t) signals pertinent to each foot from uniaxial pressure data.

### 4.1. Methods

Savelberg and De Lange [62] used an ANN to estimate each foot $GRF_{ap}\left(t\right)$ and $GRF_{ml}\left(t\right)$ signals from plantar pressure. The plantar pressure data and the corresponding GRF(t) signals were measured using a pressure insole (Novel GmbH, Munchen, Germany) and a Kistler forceplate (Kistler Instrumente AG, Winterthur, Switzerland), respectively, for a range of walking speeds (0.9–2.3 m/s) for five subjects. These data were used to train the ANN using a supervised learning method with back-propagation.

The possibilities of both inter-subject and intra-subject generalisation of ANN results were evaluated. The intra-subject generalisation referred to the possibility of training ANN with a particular walking ‘speed’ data and estimation of GRF(t) signals for other walking ‘speeds’ of the same subject. The inter-subject generalisation, on the other hand, referred to the possibility of training ANN with walking data from a particular ‘subject’ and estimate GRF(t) signals for another subject, walking at the same speed. Results of Savelberg and De Lange’s study showed a promising level of intra-subject generalisability, with a peak 10% error and mean cross-correlation coefficient of 0.9. However, the level of inter-subject generalisability was found to be inconsistent and subject/speed dependent. The ANN proposed by Savelberg and De Lange [62] was further shown to require subject- and activity-dependent training, which potentially limits its versatility and practicality.

**Figure 10:**
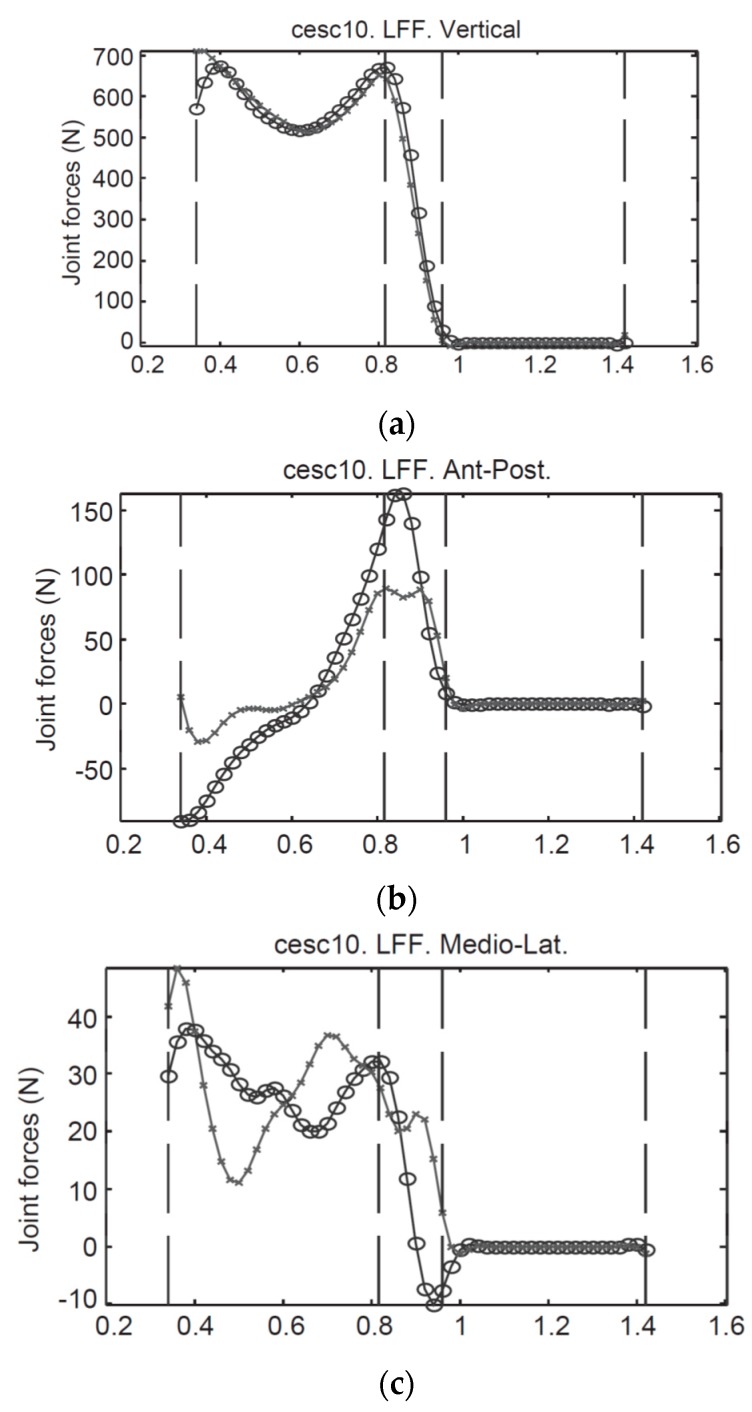
Comparison of the estimated (solid line X) vertical (**a**); antero-posterior (**b**) and medio-lateral (**c**) GRFs with the force-plates measurements (dotted line O) (after [63]).

Forner-Cordero, et al. [63] proposed using full-body kinematic data to estimate tri-axial GRF(t) signals from plantar pressure. Fifteen walking tests involving five healthy subjects were carried out and motion, ground reactions and plantar pressure data were measured using the VICON motion capture system [50], two AMTI forceplates [51] and a pair of pressure insoles, respectively. The total tri-axial GRF(t) and GRM(t) signals were calculated from the motion data using ID analysis (Equations (1)–(6)). These signals were used in conjunction with the known CoP(t) trajectories to estimate the tri-axial GRF(t) signals pertinent to each foot in such a way to satisfy the dynamic equilibrium of body forces and moments. The outputs of the model were then compared with the force plate measurements to examine their accuracy (Figure 10). The RMSE mean (standard deviation) of the estimated GRF(t) signals were found to be equal to 27.84 N (7.40 N) for $GRF_{v}\left(t\right)$, 7.53 N (1.32 N) for $GRF_{ap}\left(t\right)$ and 7.51 N (2.65 N) for $GRF_{ml}\left(t\right)$. The approach of Forner-Cordero, et al., however, was sensitive to the accuracy of the measured kinematic data, which can be challenging to achieve for long-term monitoring in outdoor environment.

**Figure 11:**
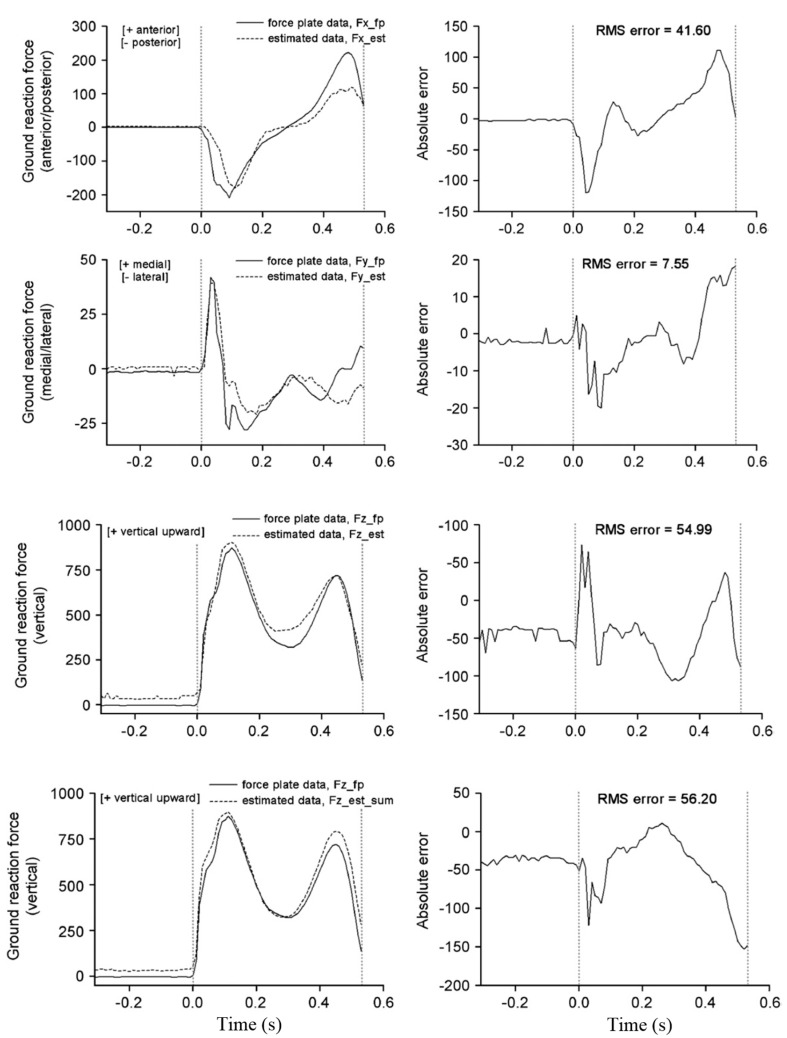
Comparison of the estimated 3D GRFs (dashed line) with corresponding forceplate measurements (solid line) (after [64]).

Fong, et al. [64] suggested using the Stepwise Linear Regressions (SLR) method [65] to estimate tri-axial GRF(t) signals from plantar pressure data. Five healthy male subjects (age: 23.0 ± 3.0 years, height: 1.72 ± 0.03 m, mass: 65.1 ± 9.7 kg, foot length: 255–260 mm) performed 10 walking trials at their natural cadence. The ground reactions and plantar pressure were measured using a force plate and a pair of pressure insoles, respectively. SLR was then used to estimate tri-axial GRF(t) signals for each foot using the pressure data. Fong, et al. [64] carried out a series of a further five walking tests and compared their results with the force plate measurements to validate their model (Figure 11). They reported the cross-correlation coefficient of 0.928 for $GRF_{ap}\left(t\right)$, 0.989 for $GRF_{v}\left(t\right)$ and 0.719 for $GRF_{ml}\left(t\right)$. The corresponding peak-to-peak normalised RMSE values were reported to be equal to 12%, 5% and 28%, respectively.

Rouhani, et al. [66] suggested a mathematical method for estimating the tri-axial GRF(t) signals from plantar pressure measurements. Eleven test subjects, six healthy (54 ± 10 years), three with ankle osteoarthritis (57 ± 19 years) and two with arthroplasty-reconstructed ankles (59 and 71 years old) participated in a series of walking tests at their preferred pace. The motion, ground reactions and pressure data were measured in each test using a VICON motion capture system [50], a Kistler forceplate (Kistler Instrumente AG, Winterthur, Switzerland) and a pair of pressure insoles (Pedar, Novel, Munich, Germany) embedded in a pair of sandals, respectively.

Both linear and non-linear mapping functions were considered to find the relationship between the measured plantar pressure and the corresponding tri-axial GRF(t) signals. The Linear Regression method was used for linear mapping and a Multi-layer Perceptron Network with one hidden layer and sigmoid basis functions [67] and Locally Linear Neurofuzzy model with an iterative training algorithm were used for non-linear mapping [68]. Two distinct strategies were used for training the mapping functions: (1) Intra-subject training, where the mapping functions were trained for a trial of a test subject and tri-axial GRF(t) signals were predicted for the other trials of the same subject; and (2) Inter-subject training, where the mapping function was trained with a trial of a test subject and the resulting functions were used to estimate the tri-axial GRF(t) signals for other subjects.

**Figure 12:**
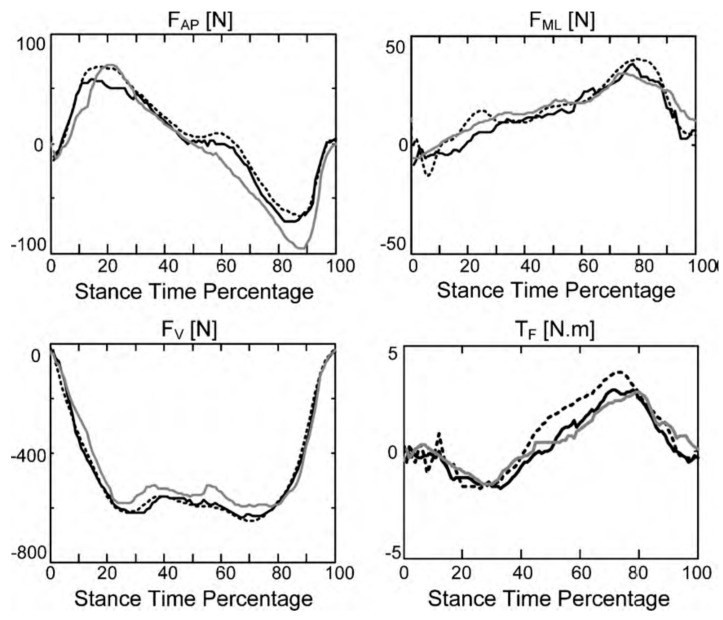
Comparison of the forceplate measurements (dashed line) with estimated anterior-posterior, medial-lateral and vertical GRFs and frictional torque for a healthy subject with intra-subject (black solid line) and inter-subject (grey solid line) training (after [66]).

The results of their study showed higher accuracy of the non-linear mapping functions (Figure 12). Lower peak-to-peak normalized RMSE were observed for $GRF_{v}\left(t\right)$ (4%) and $GRF_{ap}\left(t\right)$ (7.3%), while the $GRF_{ml}\left(t\right)$ (11.3%) and GRM(t) (14.7%) had higher errors. Furthermore, the results obtained for the healthy subjects showed less error and the intra-subject training method produced more accurate estimations.

Later in 2014, Rouhani, et al. [69] upgraded their system proposed in Rouhani, et al. [66] and included a set of IMUs (3D gyroscopes and 3D accelerometers) on the toes, forefeet, hind feet, and shanks [70] for the ambulatory measurement of multi-segment foot kinetics and kinematics during long-distance walking. A set of walking experiments were carried out on 10 healthy elderly subjects (7 females; age: 61 ± 13 years; height: 166 ± 9 cm; weight: 67 ± 10 kg) and 12 patients with unilateral ankle osteoarthritis (4 females; age: 58 ± 13 years; height: 169 ± 7 cm; weight: 81 ± 19 kg). A similar method proposed in Rouhani, et al. [66] was used to estimate tri-axial GRF(t) and CoP(t) signals. They reported peak-to-peak normalised RMSE of 10.5% and 13.7% for the sagittal and transverse ankle-joint torques in healthy subjects, respectively. The corresponding values for patients were reported to be 13.0% and 15.0%, respectively.

Jung, et al. [71] suggested two foot-ground contact models similar to those suggested by Anderson and Pandy [72] and Neptune, et al. [73] to estimate tri-axial GRF(t) signals. The Type-I model only needed the joint kinematics data to estimate tri-axial GRF(t) signals while the Type-II model needed both joint kinematics and foot pressure data.

**Figure 13:**
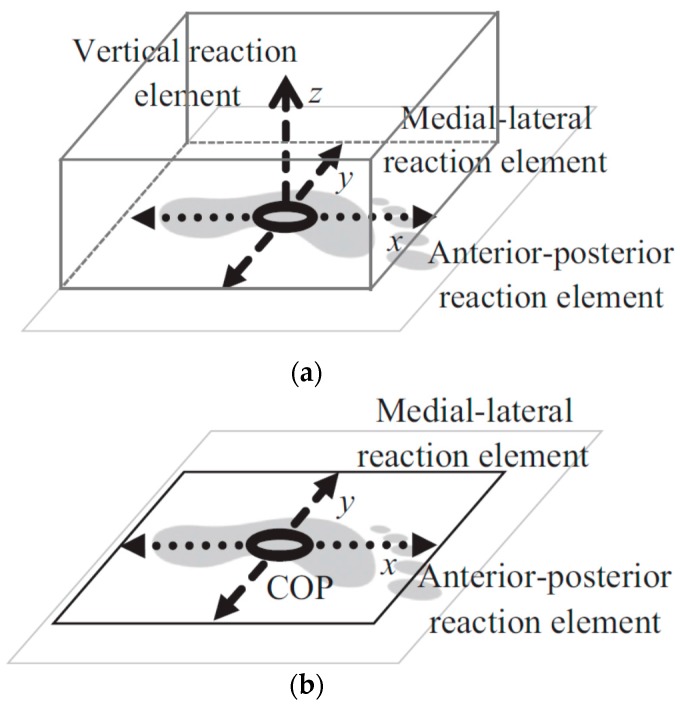
Force components of the Type-I (**a**) and II (**b**) contact models (after [71]).

The type-I model comprised 52 smart force elements that were distributed uniformly across the sole of the foot. Each force element comprised five linear reactions (Figure 13a) and its activation was controlled by a foot motion trajectory. The Type-II model, however, had only one force element, with four linear components of shear GRF(t) in medial-lateral and anterior-posterior directions (Figure 13b). This force element was placed at the CoP(t) location (found from foot pressure data) and was activated based on the total GRF(t) of the corresponding foot. The developed contact models were then integrated into the Anybody full body musculoskeletal model, with 19 segments and 38 rotational actuators [56]. The force received by each smart force element at each time-step was determined using ID-based optimisation [74].

**Figure 14:**
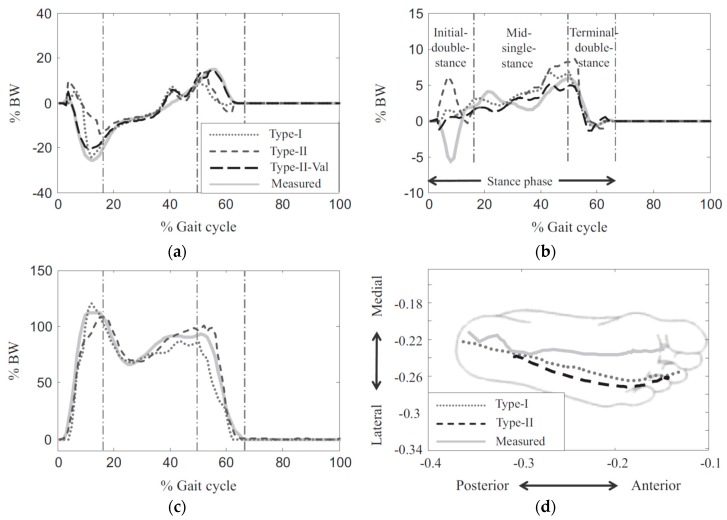
Comparison of the estimated and measured anterior-posterior (**a**), Medio-lateral (**b**) and vertical (**c**) *GRFs* and *CoP* trajectory (**d**) (after [71]).

A set of walking tests was carried out with seven healthy male subjects (age: 23 ± 2 years, weight: 67.0 ± 8.4 kg; walking speed: 1.76 ± 0.26 m/s). The motion, ground reactions and plantar pressure data were collected using a VICON motion capture system [50], two AMTI forceplates [51] and a pair of F-scan insole pressure sensors [75]. The body weight normalised RMSE mean (standard deviation) values for estimated $GRF_{ap}\left(t\right)$, $GRF_{ml}\left(t\right)$ and $GRF_{v}\left(t\right)$ signals were reported as 5.82%(5.82%), 1.87%(1.85%) and 13.75%(13.82%) for the Type-I, respectively. The error values corresponding to $GRF_{ap}\left(t\right)$ and $GRF_{ml}\left(t\right)$ signals for the Type-II model were reported as 7.82%(7.83%) and 2.67%(2.67%), respectively (Figure 14). Jung, et al. [71] models provided accurate results, however, only healthy and fit subjects were tested and the models require full body kinematic data.

Sim, et al. [76] have proposed using wavelet neural network (WNN) and principal component analysis-mutual information (PCA-MI) to estimate tri-axial GRF(t) and GRM(t) signals. They carried out a set of walking tests with three gait speeds (slow: 0.77 ± 0.06 m/s; normal: 1.14 ± 0.11 m/s; and fast: 1.73 ± 0.20 m/s) on 10 healthy subjects (seven males and three females—age: 20.6 ± 3.4 years; height: 168.2 ± 10.2 cm; and weight: 62.3 ± 8.9 kg) and 10 subjects with adolescent idiopathic scoliosis (three males and seven females—age: 17.0 ± 3.2 years; height: 158.5 ± 8.1 cm; and weight: 44.3 ± 5.0 kg). An insole-type plantar pressure measuring device (Pedar Insole, Novel GmbH, Munich, Germany) with 99 pressure sensors and two AMTI force plates [51] were used to measure plantar pressure and reference tri-axial GRF(t) and GRM(t) signals, respectively.

PCA-MI was initially used to find the input variables of the WNN model. A three-layer WNN with input, wavelet and output layers was used to estimate tri-axial GRF(t) and GRM(t) signals from pressure data. The results were then compared comprehensively with force plate measurements and a selection of methods suggested in the literature: linear regression, multilayer perceptron and locally linear neuro-fuzzy. They reported a correlation coefficient (normalised RMSE) of 0.969(12.920), 0.845(15.027), 0.976(12.957), 0.855(14.877), 0.879(18.088) and 0.836(17.880) for $GRF_{ap}\left(t\right)$, $GRF_{ml}\left(t\right)$, $GRF_{v}\left(t\right)$, $GRM_{ap}\left(t\right)$, $GRM_{ml}\left(t\right)$ and $GRM_{v}\left(t\right)$, respectively, for healthy subjects. Comparing the results of their WNN method with those of the linear regression, multilayer perceptron and locally linear neuro-fuzzy methods, they concluded that non-linear mapping functions perform better than linear models and that WNN has the highest accuracy of all considered non-linear methods.

Jacobs and Ferris [77] developed a custom instrumented insole and proposed to use ANN to estimate tri-axial GRF(t) and GRM(t) signals for walking and calf raise. In their experimental campaign, six healthy subjects (2 females, 4 males; age: 24.5 ± 3.6 years, height: 1.78 ± 0.07 m, leg length 0.94 ± 0.05 m and mass: 69.9 ± 12.64 kg) participated in a series of walking (at 1.0 m/s and 1.5 m/s) and calf raise tests. The motion data was measured using a 10 camera Vicon motion capture system [50] and ground reactions were measured using an instrumented split-belt treadmill (Bertec Inc., Columbus, OH, USA).

**Figure 15:**
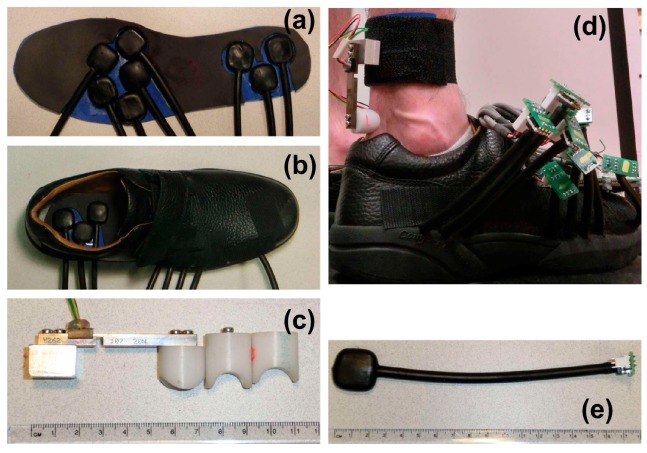
Instrumented shoe developed by Jacobs and Ferris [77].

Jacobs and Ferris developed an instrumented shoe equipped with sensory insoles (Figure 15a, b and d), each comprising eight custom neoprene bladders instrumented with miniature, amplified, temperature-compensated pressure sensors (SSCDANN030PGAA5, Honeywell, Inc., Morris Plains, NJ, USA,) (Figure 15e). Each subject also wore a miniature beam load cell on the distal end of the tibia above the calcaneus to measure localised tissue forces around the Achilles tendon (Figure 15c).

**Figure 16:**
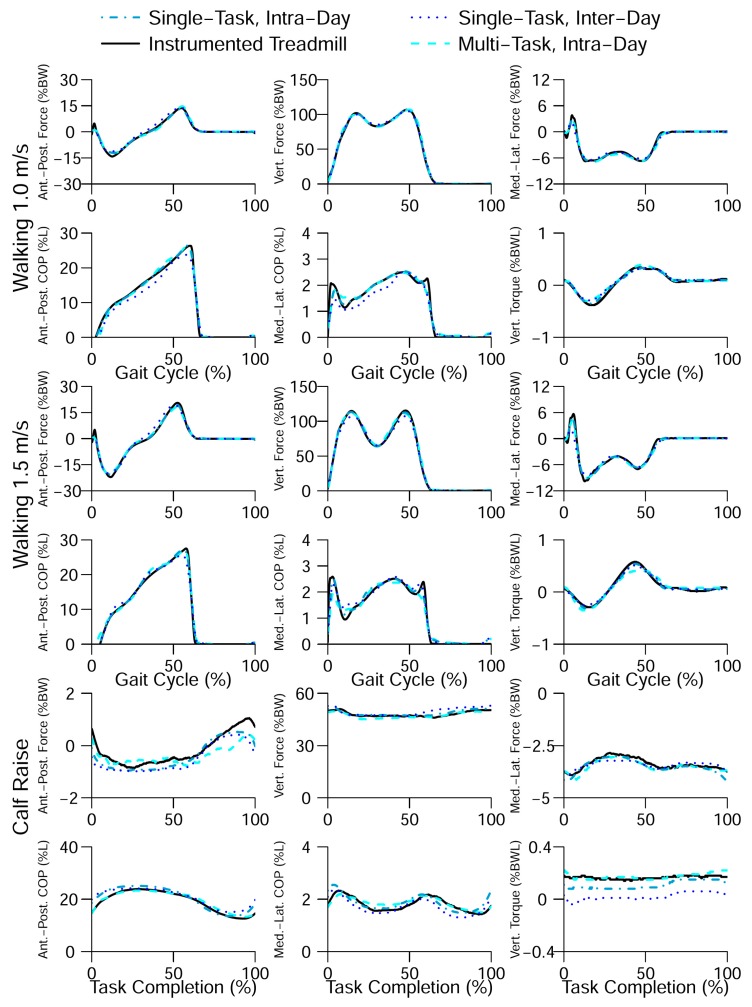
Comparison of the estimated tri-axial GRF(t) and GRM(t) signals for the method/system proposed by Jacobs and Ferris [77].

Furthermore, Jacobs and Ferris used a series of single-hidden layer, 10 node, feed-forward neural networks to estimate tri-axial GRF(t) and GRM(t) from pressure data and reported body normalised RMSE of 6.21%, 2.93%, 5.82%, 5.90% and 7.15% for $GRF_{ap}\left(t\right)$, $GRF_{v}\left(t\right)$,$\;GRF_{ml}\left(t\right)$, $CoP_{ap}\left(t\right)$ and $CoP_{ml}\left(t\right)$, respectively, for walking at 1.0 m/s (Figure 16). Results of their model trained by both walking and calf raise data resulted in normalised RMSE values of 4.15–6.80% for all three components of GRF(t) and both components of CoP(t).

### 4.2. Comparison of Methods

Table 2 compares the accuracy of the tri-axial GRF(t) signals estimated by methods suggested by Savelberg and De Lange [62], Forner-Cordero, et al. [63], Fong, et al. [64], Rouhani, et al. [66] and Jung, et al. [71] in the form of RMSE mean (standard deviation) and the cross-correlation coefficient ‘R’ between measured and estimated results. None of the suggested methods show a consistent superiority in the accurate estimation of the three components of GRF(t).

## 5. Methods Based on Tri-Axial Force Measurement

This class of methods is based on direct measurement of tri-axial GRF(t) and CoP(t) signals under each foot. As force sensors are attached to the feet and rotate with them during locomotion, the accurate measurement of sensors’ orientation is also required to transform the sensors’ measurements in their local coordinate system to the global system [61].

In the past two decades, several different force/pressure measurement technologies have been developed for ground reaction measurement such as piezoelectric [78,79], strain gauge -based [80,81], capacitive [82,83,84] and fibre optic-based sensors [85,86]. Razian and Pepper [87] developed a tri-axial force transducer using piezoelectric copolymer film that can be integrated in a shoe-sole. Hessert, et al. [88] designed a wearable force sensor based on a photo-elastic tri-axial force transducer to measure GRF(t) in gait analysis. However, only very few technologies have shown promising results for application in the field monitoring of human kinetics.

### 5.1. Methods

Chao and Yin, [89] proposed a shoe-shape force and moment measurement device comprising two tri-axial force and moment sensors placed under the heel and fore-foot of each foot. The foot complex was assumed to be a single DOF articular system with a hinge joint between the heel and toes to simulate the metatarsophalangeal articulations. They added a potentiometer at the knee joint to register the flexion or extension angle between the thigh and shank during gait. Their proposed system, however, was only able to measure tri-axial GRF(t) and GRM(t) signals in the sensors’ local coordinate system. The hinge joint between the two sensors restricted the motion of the foot to single DOF and imposed the location of centre of rotation [90]. Furthermore, no foot/sensor orientation measurement was carried out and no validation with a reference measurement data was provided.

Veltink, et al. [7,91] designed an instrumented shoe to measure tri-axial GRF(t) and GRM(t) signals using two tri-axial force and moment sensors mounted under the heel and forefoot of each foot. In their study, the orientation of the force sensors was not measured and was assumed to be the same as the ground. An optimisation procedure was used to project CoP(t) estimated using shoe data to that of the force plate. Outputs of the developed system were compared with force plate measurements for 12 walking trials of a healthy subject, and the peak-to-peak normalised RMSE of 2.3 ± 0.4% for $GRF_{v}\left(t\right)$ and RMSE of 2.9 + 0.4 mm for CoP(t) were reported. However, large deviations in $GRF_{ml}\left(t\right)$ and $GRF_{ap}\left(t\right)$ components were found, which was attributed to the errors in the estimation of foot orientation.

**Figure 17:**
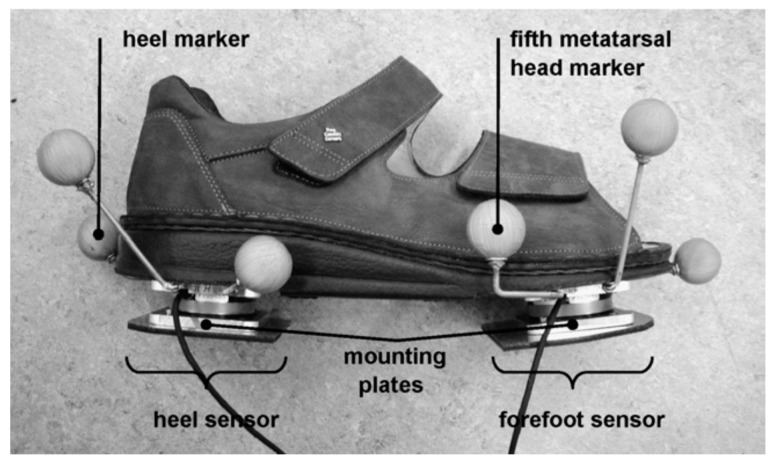
The instrumented shoe used by Liedtke, et al. [90].

Later in 2007, Liedtke, et al. [90] compared in detail the results of the instrumented shoe developed by Veltink, et al. [91] against the gold standard optical motion capture and force plate measurements (Figure 17). The results of the instrumented shoe were also compared for two types of shoe: normal weight and heavy. Seven healthy subjects (age 19–25 years) participated in a series of tests, where each subject was asked to walk across the force plate 10 times with each shoe and each foot. The instrumented shoe measurements were later transferred to the global coordinate system using the foot orientation data measured using the motion capture system.

Good agreement was reported between $GRF_{v}\left(t\right)$ measured by the instrumented shoe and the force plate (peak-to-peak normalised RMSE of 2.2 ± 0.1%). However, the results were less satisfactory for $GRF_{ml}\left(t\right)$ and $GRF_{ap}\left(t\right)$. The RMSE of 13.7 ± 2.4 mm was found for CoP(t). The most likely source of error was again identified as the errors in estimation of force sensors’ orientation. Significant difference (up to 10% of the body weight) between GRF(t) signals recorded with different types of shoes was found. However, the differences were not attributed to a specific shoe type.

Schepers, et al. [61] have updated the instrumented shoe developed by Veltink, et al. [91] by adding three miniature IMUs attached to the heel, fore-foot and shank of each foot to measure motion data. The tri-axial outputs of gyroscopes and accelerometers were combined to find the location and orientation of feet at each moment of time and to transform the force/moment sensors data into the global coordinate system. The updated system was validated against the reference motion and ground reactions data measured using an optical motion capture system and a force plate, respectively. The instrumented shoe results showed good agreement with the reference measurements except for the ankle power. The peak-to-peak normalised RMSE of 1.1 ± 0.1%, 18 ± 8% and 15 ± 5% was reported for $GRF_{v}\left(t\right)$, $GRF_{ap}\left(t\right)$ and$\;GRF_{ml}\left(t\right)$, respectively. The RMSE value of 1.1 ± 0.1% normalised to the length of shoe was reported for CoP(t). The body weight normalised RMSE of ankle moment and power were reported as 2.3 ± 0.5% and 14 ± 5%, respectively. The rather large error in the estimation of ankle power was attributed to the error in estimation of the foot motion using the reference motion capture system.

Cao, et al. [92] developed an instrumented shoe with a pair of tri-axial force sensors under the heel and forefoot of each foot to measure the tri-axial GRF(t) signals and CoP(t) under each foot. A set of walking tests were carried out on eight healthy subjects (age: 29 ± 4 years, weight: 74 ± 8.5 kg) where six IMUs were attached to the feet, shanks and thighs to measure the motion of the lower limbs. The system outputs were later used in a lower limb musculoskeletal model built in AnyBody Modelling System to estimate the lower limbs muscle forces. However, no validation against force plate measurement was provided to examine the accuracy of the ground reactions measured by their system.

**Figure 18:**
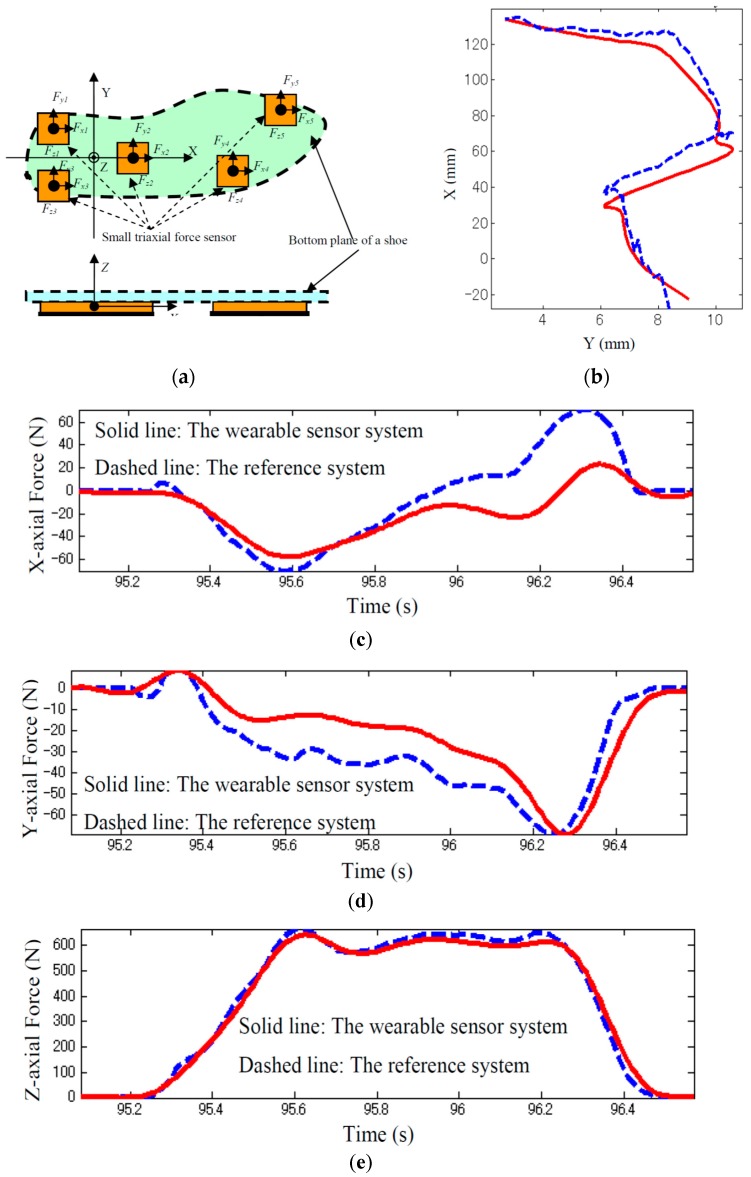
Instrumented shoe developed by Liu, et al. [95]. (**a**) Sensor layout; (**b**) CoP(t); (**c**) $GRF_{ap}\left(t\right)$; (**d**) $GRF_{ml}\left(t\right)$; (**e**) $GRF_{v}\left(t\right)$.

Liu, et al. [93,94,95] designed an instrumented shoe comprising five small tri-axial force sensors (USL06-H5-500N-C, weight: 15 g, size: 20 mm × 20 mm × 5 mm) (TEC GIHAN Co., Yokoha-city, Kanagawa, Japan) to measure tri-axial GRF(t) signals and CoP(t) of each foot (Figure 18a). A multi-channel data logger was designed to collect the data with sampling rate of 100 Hz.

To examine the performance of the system, a set of walking tests was carried out on seven young volunteers (four men and three women: age = 28.5 ± 3.5 years, height = 168.5 ± 5.5 cm, weight = 63.4 ± 9.3 kg). The measurements of a force plate and an optical motion capture system were used as a reference to validate the outputs of the instrumented shoe. No significant difference was reported between the measurements of the instrumented shoe and force plate with peak-to-peak normalised RMSE of 7.2 ± 0.8%, 9.0 ± 1% and 1.5 ± 0.9% for $GRF_{ap}\left(t\right)$, $GRF_{ml}\left(t\right)$ and $GRF_{v}\left(t\right)$, respectively (Figure 18c–e). Similarly, the RMSE of 1.4 ± 0.2%, normalised to the shoe length, was reported for CoP(t) (Figure 18b).

In 2010, Liu, et al., used a set of three low profile tri-axial force sensors (Tec Gihan Co., Yokoha-city, Kanagawa, Japan) to build a wearable force plate (weight: 86 g; size: 80 × 80 × 15 mm^3^) [96,97,98]. Two wearable force plates were used for each foot, one under the heel and one under the forefoot, to measure ground reactions. A 3D motion sensor including a tri-axial accelerometer (MMA7260Q, Sunhayato Co., Tokyo, Japan) and three uniaxial gyroscopes (ENC-03R, Murata Co. Kyoto, Japan) were attached to each wearable force plate to measure their orientation and location. The outputs of the force sensors and motion sensor were combined to calculate the tri-axial GRF(t) and CoP(t) signals in the global coordinate system.

The outputs of the proposed instrumented shoes were compared to a set of reference force plate (grounded) and optical motion capture measurements for a walking test subject. They reported peak-to-peak normalised RMSE of 5.1 ± 1.1%, 6.5 ± 1% and 1.3 ± 0.2% for $GRF_{ml}\left(t\right)$, $GRF_{ap}\left(t\right)$ and $GRF_{v}\left(t\right)$, respectively. The RMSE for CoP(t) was reported to be 3.2 ± 0.8 mm. However, the $GRF_{ml}\left(t\right)$, $GRF_{ap}\left(t\right)$ signals measured by the instrumented shoe showed an amplitude and phase shift from reference data and the discrepancy in the CoP(t) trajectory was found to be larger than the results reported by Veltink, et al. [91]. Again, they suggested the foot orientation errors as the probable source of error.

Liu, et al. [99] took another step and added four IMUs (weight: 20 g, size: 35 × 50 × 15 mm^3^), attached to the shank and thigh of each leg, to the wearable force plate system, to measure the kinematic data of the lower limbs as well. Each IMU had a tri-axial accelerometer, a tri-axial gyroscope and a tri-axial magnetometer. The motion data corresponding to each segment were calculated using a Kalman based fusion algorithm to optimally use the excellent dynamics of the gyroscope output and the stable drift-free output of the accelerometer and magnetometer. The measured data were transferred wirelessly to a computer using a portable data logger. The measured ground reactions and lower limb kinematics were later used in an ID analysis to find joint forces and torques.

**Figure 19:**
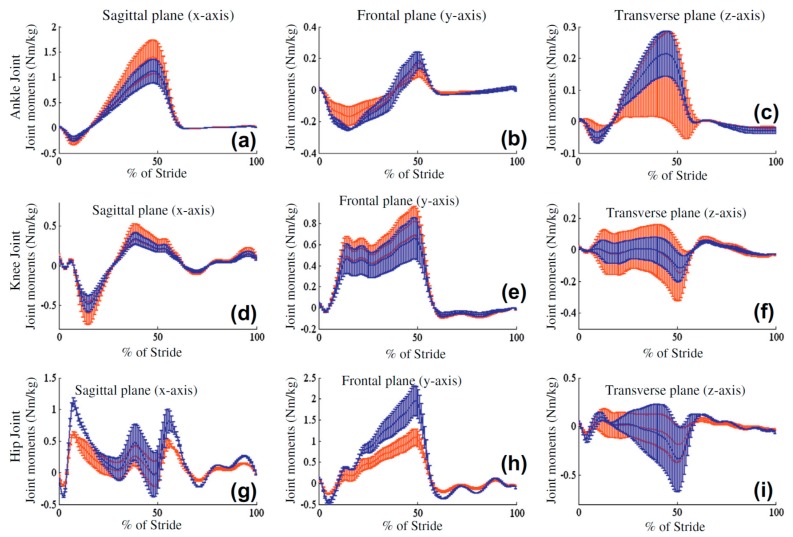
Comparison on tri-axial joint moments measured by the wearable system (red) with the reference measurements (blue) for ankle (**a**–**c**), knee (**d**–**f**) and hip joint (**g**–**i**). A standard deviation above and below the mean values are presented for the four subjects (after [100]).

The performance of this system was examined later by Liu, et al. [100] in a set of walking tests with four healthy male subjects (height: 1.62 ± 0.22 m; weight: 71.4 ± 10.1 kg). A grounded force plate (Tec Gihan Co., Yokoha-city, Kanagawa, Japan) and a Hi-DCam optical motion analysis system (NAC Image Tech., Tokyo, Japan) were used to measure the reference ground reactions and kinematic data, respectively. The measurements of the proposed system showed estimates of the ankle, knee and hip torques, with peak-to-peak normalised RMSEs of 7.1%, 13.4% and 21.0%, respectively (Figure 19).

Adachi, et al. [101,102,103], used an almost identical system to Liu, et al. [99] to measure ground reactions and kinematic data using a combination of wearable force plates and IMUs. The ground reactions were measured using two pairs of wearable force plates attached under the heel and forefoot of each foot. The motion data were measured using six tri-axial IMUs attached to the shanks, thighs and lower and upper back. Each wearable force plate also had a similar IMU to measure its motion. A set of walking tests was carried out on a healthy male subject (age: 23 years, weight: 62 kg, Height: 170 cm) and the reference motion and ground reactions data were measured using an optical motion capture system (Eagle Digital Camera: Motion Analysis) and two AMTI force plates [51], respectively. The maximum peak-to-peak normalised errors of 6.4% and 4.7% were reported for GRF(t) and CoP(t), respectively.

Lincoln, et al. [104] used five optical tactile sensors embedded in an elastomeric insole to measure the tri-axial GRF(t) signals. Each sensor is composed of a reflective/absorptive surface, a layer of transparent material, five light sources (emitter) and five light sensors (detector). When a normal load is applied to the sensor, the interstitial transparent material compresses and the reflective martial moves closer to the emitter and detector. Shear loads are measured by sensing the ratio of absorptive to reflective material between the emitter and detector. A linear least squares regression is used to train the model that relates the sensor’s measurements to each component of GRF(t) signals.

**Figure 20:**
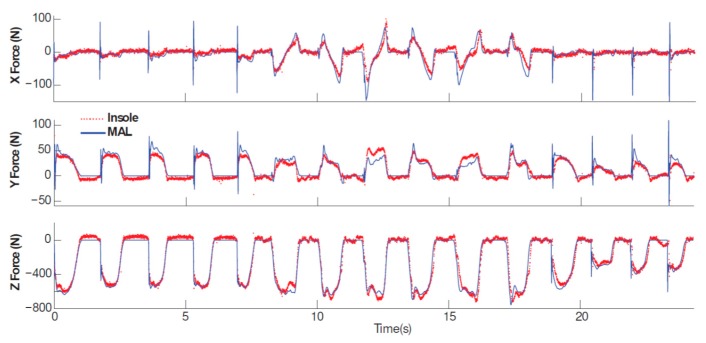
Comparison of A-P (X), M-L (Y) and vertical (Z) GRF measured by insole (red dots) with corresponding force-plate data (blue line) (after [104]).

A series of five forward and backward walking tests were carried out on a single test subject. To validate the insole measurements, foot motion and ground reactions were also measured using a VICON motion capture system [50] and an AMTI OR6-7-2000-TT force plate, respectively. The mean and standard deviation of the error values (averaged over five trials) were reported as 13.75 ± 22.94 N, 7.71 ± 10.99 N and 68.10 ± 107.18 N for $GRF_{ap}\left(t\right)$, $GRF_{ml}\left(t\right)$ and $GRF_{v}\left(t\right)$, respectively (Figure 20). Although the proposed sensor claimed to be inexpensive and practical to use, its accuracy might not be enough for some medical applications. Moreover, the results of optical tactile sensors were shown to be temperature dependent and require complex training models to include their non-linearity and hysteresis effects.

### 5.2. Comparison of Methods

Table 3 compares the RMSE mean and standard deviation in estimation of GRF(t) and CoP(t) signals using methods suggested by Veltink, et al. [7], Veltink, et al. [91], Liedtke, et al. [90], Schepers, et al. [61], Liu, et al. [105], Liu, et al. [95] and Liu, et al. [98]. The method suggested by Liu, et al. [105] shows the highest accuracy in the estimation of $GRF_{ml}\left(t\right)$, while $GRF_{ap}\left(t\right)$ and $GRF_{v}\left(t\right)$ signals estimated by Liu, et al. [95] model have the least error.

## 6. Discussion

**Figure 21:**
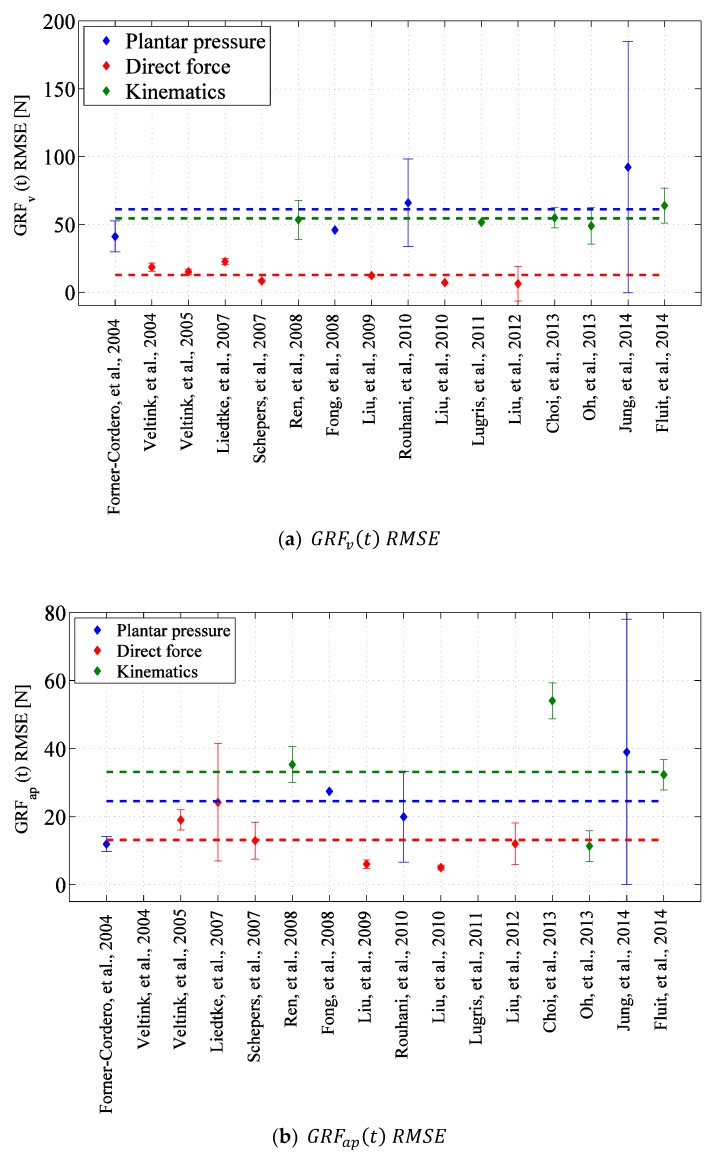

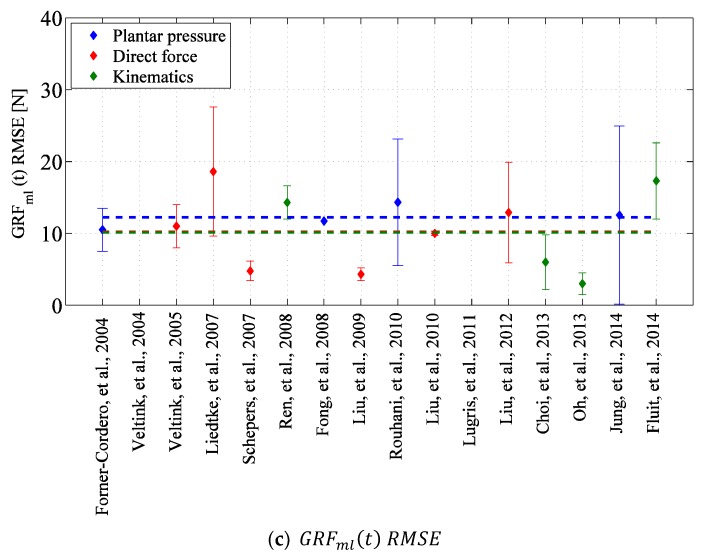
Comparison of the mean standard deviation of the RMSE of the estimated (**a**) $GRF_{v}\left(t\right)$; (**b**) $GRF_{ap}\left(t\right)$ and (**c**) $GRF_{ml}\left(t\right)$ signals for different methods. The average of mean values for each class of methods is shown with a dashed line with corresponding colour.

The *accuracy* of the estimated GRF(t), GRM(t) and CoP(t) signals for all three classes of methods are cross-compared in Figure 21 using the reported RMSE mean and standard deviation values (the Savelberg and De Lange, [62] study is not shown in Figure 21 due to its inadequate information. Furthermore, SRSS method is used to calculate the overall error in studies where the accuracy of the left and right foot GRF(t)s are reported separately). In this figure, the average of mean RMSE values for each class of method are shown using a dashed line with corresponding colour. According to Figure 21, the methods based on measured kinematic data, plantar pressure and direct GRF(t) measurement estimated $GRF_{v}\left(t\right)$ with average RMSE mean (standard deviation) error of 54(12) N, 61(45) N and 13(3) N, respectively. The corresponding error values for $GRF_{ap}\left(t\right)$ are 33(5) N, 25(18) N and 13(6) N and for $GRF_{ml}\left(t\right)$ are 10(3) N, 12(8) N and 10(4) N, respectively. Comparing the mean RMSE values, overall, the results of the methods that directly measure ground reactions show slightly higher accuracy compared with the methods that estimate tri-axial GRF(t) and CoP(t) signals using measured body kinematics and plantar pressure. However, all three classes of methods show competitive accuracy levels.

The key sources of errors in estimating GRF(t), GRM(t) and CoP(t) signals for each class of methods are:

- Kinematics-based methods: (1) these methods rely on a dynamic human model to estimate GRF(t), GRM(t) and CoP(t) signals. It has been shown that the accuracy of the estimated GRF(t) signals is very sensitive to the characteristics of the human model such as foot [106] and knee joint [107] models. This can be a source of significant uncertainty in the accuracy of the model outputs; (2) errors in measured kinematic data, particularly errors in the measured orientations in the case of using wearable IMUs [108]; (3) the simplifying assumptions used in body dynamic model and the inverse dynamics analysis, such as solid body segments and frictionless joints; (4) the inaccuracies in anthropometric data, particularly the size, density and weight of body segments, the location of joint centres and the location of centre of mass of each body segment; (5) soft tissue artefacts (STA) [109,110]; and (6) inherent computational errors of the methods proposed to solve the indeterminacy problem of the closed-kinematic chain during DSP.
- Methods based on measurement of Plantar pressure: (1) the low accuracy and rapid deterioration (resulting in time varying calibration) of the pressure sensors; (2) high sensitivity of the insole pressure sensors to their boundary conditions in the shoe [111]; and (3) the errors associated with the estimation of tri-axial GRF(t) signals from uniaxial plantar pressure data.
- Methods based on direct measurement of GRF(t): (1) errors associated with estimating forces and moments in the coordinate systems of body segments and joints, because of uncertainties in relative positions and deformation of segments, especially of the foot [91]; (2) errors associated with IMU orientation measurement as instrumented shoes use IMUs to measure the orientation of each sensor with respect to the global/body coordinate system.

From a *practicality* point of view, compared with the methods that estimate tri-axial GRF(t) and CoP(t) signals using pressure insoles and force sensors, methods based on measuring body kinematics using IMUs require smaller data acquisition system. Considering that IMU sensors are cheaper in price, smaller in size, more durable and lower in power demands compared with pressure insoles and instrumented shoes, they seem to be a very appealing option for long-term real-life measurement. However, prohibitively large number of IMUs required to capture full body motion, hinders their practicality for long-term use. Developing methodologies to estimate tri-axial GRF(t) and CoP(t) signals using a limited number of IMUs and optimizing their location on the body to achieve both maximum accuracy and practicality is an important avenue that merits future research.

The other important aspects that merit further consideration are:

- *Versatility and Robustness*: many of the discussed methods are only validated for a particular movement [36,37] or methodologies are developed based on a limited dataset [38,39] that may not be applicable for movements other than those present in the dataset. To be able to use the proposed methodologies in real-life setting, it is important for the method to be robust and versatile enough to handle different movements and ambulation abnormalities. Future proposed methods could try to analyse the performance of the method for different ambulatory regimes, pathological gaits and physical environment conditions such as slopes, slippery surfaces, etc., and to provide experimental validation for such scenarios. For instance, knowing that real-life meausrement entails monitoring not only walking but several different activates such as sitting, turning, running, etc., combining a set of activity-specific GRF(t) estimation methods with and activity recognition method to link the type of activity with corresponding GRF(t) estimation method could be a possible direction for tackling this problem.
- *Training requirement*: Due to the inter- and intra- subject variability of human gait, many of the GRF(t) estimation methods, including the ones based on Artificial Neural Networks [26,48], rely on training data for calibration. However, such training data might not be available. Therefore, it is desirable for the methodologies not to require training data or to provide a generic form that works with reasonable accuracy for the cases where training data is not available. Considering the potential benefits of personalizing the parameters of GRF(t) estimation methods, an interesting avenue for the future research could be to develop methodologies that autonomously self-train and personalize their parameters using only the available sensors of the system.
- *System design*: Minimizing the size and weight of the sensors and data acquisition systems, reducing their power demand and increasing their battery life, particularly through methods such as energy harvesting from ambulation [112] are fundamental to the application of these systems for long-term monitoring.

In the future research in this area, in addition to reporting the overall accuracy of the estimated signals using RMSE which represents the standard deviation of the differences between predicted values and observed values, it is recommended that the performance of the proposed methods would be analysed and validated in terms of variability (using statistical measures such as variance and standard deviation), repeatability (using statistical measures such as inter- and intra- class correlation coefficients and confidence intervals [113], global and regional Person correlation coefficient, coefficient of variation [114] and coefficient of multiple correlation [114]), reproducibility (using statistical measures such as *t*-test [115], analysis of variance (ANOVA) [115], inter- and intra- class correlation coefficients, standard error of measurements and smallest detectable difference [116]), specificity and sensitivity [117]. Furthermore, the estimated GRF(t) and CoP(t) signals are recommended to be analyzed in terms of representing spatial (e.g., stride length, foot clearance, etc.) and temporal (step duration, swing and stance phase timing, etc.) gait metrics and parameters such as cycle-by-cycle peak GRF(t) magnitude. Finally, detailed evaluation of other technical and practical aspects of the proposed methodologies such as their computational demand and their suitability for ‘real-time’ application is highly recommended.

## 7. Conclusions

This paper reviewed the techniques and technologies proposed in the literature to estimate or measure tri-axial GRF(t), GRM(t) and CoP(t) signals of a walking human outside a laboratory environment. Three classes of methods were reviewed based on the type of measured data: (1) methods based on measured kinematic data; (2) methods based on measured plantar pressure; and (3) methods based on directly measured ground reactions. Comparing the results of the proposed methods, it was concluded that the methods based on the direct measurement of ground reactions have the highest accuracy and the least practicality for long-term real-life monitoring applications, whereas methods based on measured kinematic data have the lowest accuracy but the highest practicality of the three classes.

None of the reviewed methods, even methods based on the kinematic data, are well-developed and extensively validated for long-term measurement application in a real-life environment. The methods based on measured kinematic data usually rely on full-body kinematic measurement. This entails using a prohibitively large number of sensors, which makes it impractical for long-term use. On the other hand, the pressure insole sensors employing ‘force sensing resistor’ technology are often characterised by low accuracy, rapid deterioration, short lifetime and high sensitivity to their boundary conditions in the shoe. These issues again significantly limit their application for long-term measurement.

Finally, wearable force plates are expensive and cumbersome and require a large power source and data acquisition system. Instrumenting shoes with force sensors and IMUs, not only poses a serious practicality issue, but also considerably changes the contact mechanism between the foot and ground, affecting the natural gait parameters.

Overall, reducing the size and price of tri-axial load-cells, improving the accuracy of orientation measurement using IMUs, minimizing the number and optimizing the location of required IMUs for kinematic measurement, increasing the durability of pressure insole sensors, and enhancing the robustness and versatility of the ground reactions estimation methods to include pathological gaits and natural variability of gait in real-life physical environment are among the key directions that merit future research.

## Figures and Tables

**Table 1:** Comparison of the mean (standard deviation) of RMSE for the methods suggested by Ren, et al. [39], Lugris, et al. [46], Choi, et al. [48], Oh, et al. [26] and Fluit, et al. [55].

Signal		Error	Ren, et al. [39]	Lugris, et al. [46]	Choi, et al. [48]	Oh, et al. [26]	Fluit, et al. [55]
GRF(t)	$GRF_{v}\left(t\right)$	RMSE (N/kg)	0.71(0.19)	0.687(-)	0.73(0.10)	0.65(0.18)	0.85(0.17)
		NRMSE (%)	5.6(1.5)	-	5.68(1.80)	5.8(1.0)	6.9(1.3)
	$GRF_{ap}\left(t\right)$	RMSE (N/kg)	0.47(0.07)	-	0.72(0.07)	0.15(0.06)	0.43(0.06)
		NRMSE (%)	10.9(0.83)	-	13.2(2.21)	7.3(0.8)	8.5(1.6)
	$GRF_{ml}\left(t\right)$	RMSE (N/kg)	0.19(0.03)	-	0.08(0.05)	0.04(0.02)	0.23(0.07)
		NRMSE (%)	20.0(2.7)	-	12.5(4.74)	10.9(1.8)	16.6(4.6)
GRM(t)	Sagittal	RMSE (Nm/kg)	0.20(0.11)	0.241(-)	-	0.08(0.05)	0.17(0.07)
		NRMSE (%)	12.2(4.8)	-	-	9.9(1.9)	10.4(3.7)
	Frontal	RMSE (Nm/kg)	0.15(0.01)	0.154(-)	-	0.05(0.03)	0.13(0.03)
		NRMSE (%)	32.5(4.3)	-	-	22.8(4.9)	27.1(9.0)
	Transverse	RMSE (Nm/kg)	0.04(0.02)	-	-	0.03(0.02)	0.28(0.08)
		NRMSE (%)	26.2(9.4)	-	-	25.5(4.5)	38.4(10.9)

**Table 2:** Comparison of the RMSE mean (standard deviation) and cross-correlation coefficient of the methods suggested by Savelberg and De Lange [62], Forner-Cordero, et al. [63], Fong, et al. [64], Rouhani, et al. [66] and Jung, et al. [71].

Signal	Error	Savelberg and De Lange, [62]	Forner-Cordero, et al. [63] Right Foot Left Foot		Fong, et al. [64]	Rouhani, et al. [66] Inter-Subject	Jung, et al. [71] Type-I
$GRF_{v}\left(t\right)$	RMSE (N)	-	27.84(7.40)	30.13(8.70)	45.79	65.90(35.25)	92.13(92.59)
	R (N)	-	0.997	0.995	0.989	0.970(0.038)	-
$GRF_{ap}\left(t\right)$	RMSE (N)	-	7.53(1.32)	9.15(1.80)	27.41	19.93(13.36)	38.99(38.99)
	R (N)	Median: 0.879 Range: 0.621–0.963	0.979	0.977	0.928	0.976(0.017)	-
$GRF_{ml}\left(t\right)$	RMSE (N)	-	7.51(2.65)	7.30(1.48)	11.71	14.32(8.82)	12.53(12.40)
	R (N)	-	0.818	0.778	0.719	0.812(0.195)	-

**Table 3:** Comparison of the RMSE mean (standard deviation) in estimation of tri-axial GRF(t) and CoP(t) signals using methods suggested by Veltink, et al. [81], Veltink, et al. [82], Liedtke, et al. [80], Schepers, et al. [51], Liu, et al. [96], Liu, et al. [86] and Liu, et al. [89].

Method		$GRF_{v}\left(t\right)$ RMSE (N)	$GRF_{ap}\left(t\right)$ RMSE (N)	$GRF_{ml}\left(t\right)$ RMSE (N)	CoP(t) RMSE (mm)
Veltink, et al. [7]		18.4(3.1)	-	-	3.1(0.4)
Veltink, et al. [91]		15.0(2.0)	19.0(3.0)	11.0(3.0)	2.9(0.4)
Liedtke, et al. [90]		22.5(2.1)	24.2(17.3)	18.6(9.0)	9.9(1.8)
Schepers, et al. [61]		8.16(0.86)	12.92(5.44)	4.76(1.36)	5.1(0.7)
Liu, et al. [105]		12.1(1.1)	6.0(1.3)	4.3(0.9)	3.2(0.8)
Liu, et al. [95]		7.0(0.4)	5.0(0.7)	10.0(0.3)	2.1(0.4)
Liu, et al. [98]	Left foot	4.95(3.25)	7.5(2.7)	3.67(3.29)	-
	Right foot	3.74(12.35)	9.35(5.46)	12.4(6.23)	-

## Nomenclature

$a_{i}$	Linear acceleration of the centre of mass of body segment ‘*i*’
$\alpha_{i}$	Angular acceleration around the centre of mass of the body segment ‘*i*’
ADL	Activities of daily living
ANN	Artificial neural network
BMI	Body mass index
CoM	Centre of mass
*CoP*	Centre of plantar pressure
DSP	Double-support phase
DoF	Degree of Freedom
FCM	Foot-ground contact model
*GRF*	Ground reaction force
$GRF_{v}$	Ground reaction force in the vertical direction
$GRF_{ap}$	Ground reaction force in the anterior-posterior direction
$GRF_{ml}$	Ground reaction force in the medial-lateral direction
*GRM*	Ground reaction moment
$I_{i}$	Second moment of inertia of the body segment ‘*i*’
ID	Inverse dynamics
IMU	Inertial measurement unit
$k_{1}$ and $k_{2}$	Empirical constants
$m_{i}$	Mass of the body segment ‘*i*’
$n_{s}$	Number of (solid) body segments
NRMSE	Normalised root mean square error
PCI-MI	Principal component analysis—mutual information
R	Cross-correlation coefficient
$r_{i}$	Moment arm pertinent to the body segment ‘*i*’
RMSE	Root mean square error
SLR	Stepwise linear regression
SSP	Single-support phase
$T_{ds}$	Half of the double-support duration
WNN	Wavelet neural network
